# Evaluation of the potential of grape vinegar pomace as adsorbent for Hg(II) removal

**DOI:** 10.1038/s41598-025-24665-6

**Published:** 2025-11-19

**Authors:** Ezgi Kınalı, Deniz Bingöl

**Affiliations:** Faculty of Science and Arts, Department of Chemistry, Kocaeli University, 41001 Kocaeli, Turkey

**Keywords:** Adsorption, Grape vinegar pomace, Hg(II), RSM, Chemistry, Environmental sciences, Materials science

## Abstract

In this study, the efficiency of grape vinegar pomace (GVP), an agricultural waste product, for Hg(II) removal from aqueous solutions was investigated for the first time. The conventional method (one factor at a time, OFAT) was used to examine the effects of pH, contact time, adsorbent amount and initial Hg(II) concentration on Hg(II) adsorption by GVP. The adsorption process was endothermic, spontaneous, and had an increasing entropy, according to thermodynamic analysis, while kinetic analysis revealed that the rate-limiting step is in line with the pseudo-second-order model. The most appropriate model for adsorption equilibrium, the Langmuir isotherm model, demonstrated that the adsorption process took place as a monolayer over homogeneous surfaces. For experimental optimization, the response surface methodology (RSM) approach was also used to describe the adsorption process. The central composite design (CCD) method was used to design the trials in order to examine the effects of the amount of adsorbent and the initial concentration of Hg(II). With an initial Hg(II) concentration of 150 mg/L and an adsorbent amount of 0.09 g, the highest removal efficiency of 96.2% was achieved. GVP has the potential to be a low-cost, easily available, highly effective, and ecologically friendly wastewater alternative source because of its rapid removal effectiveness.

## Introduction

Heavy metal ions originating from industrial discharges are hazardous due to their high toxicity effects. Mercury contamination, especially from industrial activities, has serious effects on the environment, human health, and ecosystems. The presence of mercury(II) or Hg^2+^ is a significant threat to humans, aquatic life, and plants, and the potential health risks associated with mercury cover a wide range of toxic effects, most of which are usually irreversible^1,2^. There are three main types of mercury: elemental (Hg(0)), organic (methylmercury or ethylmercury), and inorganic (Hg(I) and Hg(II) or Hg^2+^). All three forms of mercury are hazardous and can cause health problems; however, metallic (Hg^2+^) and organic forms are considered to be the most toxic forms for living organisms. Exposure to mercury can occur through skin absorption, inhalation, and ingestion^3^. Despite the fact that organic mercury is more dangerous than inorganic ionic mercury due to its lipophilia, inorganic mercury (II) is frequently found in natural aquatic environments and industrial wastewater due to its high solubility and stability^4^. Central nervous system dysfunction, muscle weakness, vision loss, skin rashes, mobility disorders, memory problems, speech difficulties, and hearing problems are all possible outcomes of mercury poisoning. When mercury gets into water, fish naturally change it into methylmercury (CH_3_Hg^+^), which is extremely harmful. Fish, fish-eating animals, and predators that consume fish-eating animals can develop “Minamata disease” due to the buildup of methylmercury in these organisms. Mercury pollution is mostly caused by Hg^2+^ emissions from mining, industrial production, and fossil fuel combustion. Effective management of the Hg^2+^ concentration in these emissions is necessary to limit the amount of this pollutant released into the environment and to avoid negative impacts on ecosystems and human populations. Similar to many other pollutants found in drinking water, this element can be dangerous at concentrations or levels that don’t change the water’s appearance, taste, or smell. Environmental harm can be irreversible if mercury is released in excess of the threshold for Hg²⁺ concentrations in wastewater^5^. Because of this, the World Health Organization (WHO) and the United States Environmental Protection Agency (US EPA) have listed Hg^2+^ as a priority pollutant. The WHO has established guidelines of 1 µg/L for drinking water and 5 µg/L for wastewater to reduce the negative impacts of Hg^2+^ on the environment and public health. The US EPA has published a limit of 2 µg/L for drinking water and 10 µg/L for wastewater containing Hg^2+^^6–8^. These restrictions must be met by facilities that release industrial wastewater containing Hg^2+^ into sewers. Thus, Hg^2+^ removal from industrial wastewater has been the cornerstone of many research articles^5,9^. It is crucial to develop an effective method to remove Hg(II) from water. There are various physical, chemical and biological methods in the literature for the removal of Hg(II) from industrial wastewater. Therefore, many investigators are trying to develop technologies that may decrease and control the negative effects of Hg^2+^. Many methods have been used over the years to extract Hg^2+^ from water, including ion exchange, extraction, membrane filtration, chemical precipitation, and most notably adsorption^10^. Although the ion exchange method is more expensive and sensitive to impurity presence, it provides better metal selectivity and regeneration performance. Despite being simple and cost-effective, the chemical treatment method responds slowly and often produces hazardous byproducts and secondary water contamination. The electrolysis method can remove mercury pretty effectively, but it is costly and energy-intensive and can also produce mercury vapour. Even though the membrane processing method efficiently removes a sufficient number of mercury (II) ions, its widespread application is limited by low selectivity, instability in the presence of pollution, high pressure, and high concentrations of mercury ions^11^. However, because of its excellent efficacy, low operating cost, and simplicity in design and operation, the adsorption technique is often regarded as the most effective heavy metal treatment method. When using a biomass-based adsorbent, the adsorption method has proven to be one of the most promising strategies for the removal of mercury (II) when compared to other ways^4^. The use of bio-adsorbents has several advantages, including economic feasibility, a large variety of available raw materials, cost-effectiveness, ease of use, selective removal ability, and good removal efficiency and adsorption capacity. Utilizing biomass pomace to create an adsorbent can lower the cost of water treatment^1^. These simply require minor pretreatment, such as washing, drying, grinding, or a minor acid or alkali treatment, therefore no substantial physical or chemical treatments are required^12^. Agricultural pomaces frequently contain polar functional groups, including cellulose, lignin, alcohols, aldehydes, ketones, carboxylic, phenolic, and ether groups^13^. The broad harmful consequences of diverse emissions from industrial processes on the environment have emerged as a global issue, attracting the attention of several stakeholders and experts. Furthermore, the most appropriate technique for removing heavy metals from water/wastewater is adsorption, and the use of natural adsorbents has become a preferred alternative for many researchers^10^. Agricultural pomaces such as pistachio shells^14^, palm shells^15^, bamboo leaves^16^, corn straw^17^, coffee pomace^18^, grape bagasse^19^, greengrape marc^20^ and pandanus leaves (pandanus tectorius)^21^ have been used in various forms for aqueous Hg^2+^ adsorption. These pomaces have the potential to be a low-cost, high-efficiency, and environmentally friendly alternative source. Therefore, the search for new lower cost materials in water treatment processes will continue to be examined as an important issue^22^. The major raw materials used in the food processing sector are fruits and vegetables. Pomace, also known as pulp (if processed for juice or oil), is typically produced in vast amounts after processing and poses a significant economic and environmental burden on both the industry and society. Although the precise amount of food pomace produced each year is unknown, it is commonly acknowledged that food pomace is a major problem that needs to be addressed and reduced. Therefore, the industry is constantly looking for new applications for these materials^23^. Vegetable- and fruit-derived adsorbents have been widely used for the removal of toxic metals from aqueous environments. They offer several advantages, such as being abundant, low-cost, and, in some cases, more efficient than synthetic adsorbents. Their greenness is expressed in two ways: they are less toxic than synthetic adsorbents, and pomace materials from vegetables and food can be used for environmental remediation^24^. For example, grapes are one of the most valuable traditional fruits in the world. It can be consumed raw or used to make wine, jam, juice, jelly, raisins, vinegar, and vegetable oil. After fermentation, the wine industry generates millions of tons of trash (grape pomace), which raises the issue of pulp management on both an environmental and economic level. After the grapefruit juice is extracted, the grape pomace, which contains the stem, seed, and skin of the grape, emerges^25,26^. Inappropriate disposal of grape pomace can have detrimental effects on the ecosystem. Due to expensive fees and transportation costs, wineries frequently have few options for disposal. Alternative uses for these pomaces should be investigated, even if studies on safe and economically feasible chemical disposal have been done. Food waste should be recycled because it is costly and harmful to the environment. With its substantial lignocellulosic content, grape pomace has a great deal of promise for the creation of sustainable and value-added goods^23^.

Classical/conventional methods are unable to display every possible combination of factors influencing the process. However, testing these techniques to find the ideal levels takes a lot of effort. On the other hand, experimental design including response surface method (RSM) approach is a good way to build, improve and optimize the process. This technique also investigates the interrelationship between various factors for optimum conditions of the process which helps to determine the interactions between the optimized parameters. RSM’s main objective is to identify the system’s ideal operating circumstances or an area that balances the operational characteristics^27,28^.

In this study, an investigation was made on the adsorption of Hg(II) ions in a water environment on a low-cost adsorbent obtained especially from natural and industrial pomace materials. Grape vinegar pomace was selected as a novel and appropriate adsorbent that has not yet been evaluated in this field. Adsorption isotherms, kinetic and thermodynamic variables evaluated from adsorption equilibrium data, as well as the effects and interactions of adsorption variables, were investigated with RSM.

## Materials and methods

### Materials and experiment apparatuses

In this study, the removal mechanism of Hg(II) ions from water/wastewater by the batch adsorption method, equilibrium relationships, and the use of response surface methodology (RSM) for experimental optimization were comprehensively investigated in addition to the identification of the nature of adsorption. Grape vinegar pomace (GVP) was used as a new low-cost adsorbent for the removal of Hg(II) from aqueous solutions. GVP samples were provided by a local vinegar mill in Kocaeli. The pomace materials were washed several times with ultrapure water and then dried in an oven (~ 48 h) at 333 K. They were then ground, sieved, and dried again at 333 K (~ 24 h) and stored in a desiccator for experimental studies. For the purpose of this study, only inorganic aqueous mercury (Hg(II) or Hg^2+^) was investigated. A stock Hg(II) solution (1 g/L) was prepared using Merck’s mercury (II) acetate (Hg(CH_3_COO)_2_, MW: 318.68 g/mol) (EMSURE^®^ ACS for analysis). To prepare standard and working solutions at the appropriate concentrations, the stock solution was diluted with ultrapure water (18.2 MΩ) before to each experiment (Elga PURELAB Flex 1).

Adsorption experiments were carried out in 50 mL amber bottles using 25 mL of Hg(II) acetate solutions at the indicated concentrations (*C*_o_, mg/L) and the indicated amounts (*m*, g) of GVP. The bottles were shaken at a constant speed of 200 rpm using an orbital shaker (N-Biotech Orbital Shaker/NB-101 S) for the desired contact times at room temperature. The temperature effect was investigated in kinetic studies; however, adsorption experiments were conducted at room temperature (298 K).

Hg(II) analyses were performed using the “diphenylcarbazide method” using 0.1% 1,5-diphenylcarbazide solution with a UV-visible spectrophotometer (T80 + UV-VIS, PG Instruments Ltd.) using a calibration graph at *λ*_max_ (534 nm)^29^. Solution pH measurements were carried out using a HANNA/pH 211 model pH meter. pH adjustments of the solutions were made using 0.1 M NaOH and 0.1 M HCl solutions.

Methods such as Fourier Transform Infrared Spectrophotometer (FTIR, Perkin Elmer 400/Bruker IFS 66/S) were used to determine the chemical shift and functional groups; Scanning Electron Microscope (SEM, QUANTA 400 F and a JEOL JSM-6060) to observe surface morphology, and Brunauer-Emmett-Teller (BET, Quantachrome Corporation, Autosorb-6) for surface area and pore size analysis was used. These analyses were used to elucidate the adsorption mechanism by investigating the effects of physical and chemical properties of the GVP before and after adsorption on Hg(II) removal. Also, thermal analysis methods were used to investigate the thermal stability of GVP. TGA-DTA analysis (Setaram Labsys TGA-DTA) was carried out simultaneously in the temperature range of 25–900 °C with a heating rate of 10 °C/min in a nitrogen atmosphere.

pH shift method was used to determine the point of zero charge (pH_pzc_). The adsorbent’s capabilities are determined by its surface charge and pH_pzc_. One of the isoelectric kinetic characteristics of materials is the isoelectric point, which is pH_pzc_. This refers to the pH value at which the surface of a material reaches a neutral state under certain pressure and temperature conditions. The pH at pH_pzc_ is defined as the pH value of zero net charge density on the surface of the adsorbent^30^. Determination of the pH_pzc_ value aims to determine the pH value at which the surface of the biosorbent carries an equal number of negative and positive charges (zero net charge). pH_pzc_ is important in surface characterization as it controls the rate of absorption of potentially hazardous ions by the adsorbent.

In this study, the method’s accuracy and reliability were also interpreted using response surface methodology (RSM) as a statistical technique. The effects of two independent variables (analyte concentration and adsorbent amount) on the response functions (*R*% and *q*_e_) were investigated using RSM with central composite design (CCD), and optimum conditions were determined by maximizing Hg(II) removal percentage and adsorbent capacity.

### Adsorption performance of GVP for Hg(II) removal

Adsorption performances for GVP were evaluated by batch adsorption experiments. For the removal of Hg(II) from aqueous solutions, parameters such as initial solution pH, initial Hg(II) concentration, adsorbent amount, contact time and temperature were investigated. Kinetic models were applied to the experimental data to determine the adsorption mechanism, and isotherm models were applied to describe the system at equilibrium. In addition, thermodynamic parameters were calculated to describe the nature of adsorption. An adsorption isotherm gives the equilibrium relationship to describe the interaction between adsorbate and adsorbent at a constant temperature when both adsorption and desorption occur at the same rate. Equilibrium isotherm modelling was performed to determine the best-fitting model to the experimental data among various equilibrium isotherm models (Langmuir, Freundlich, and Temkin). This modelling gives an idea about the adsorbent capacity, surface properties, and adsorption mechanism. Since a high adsorption rate is an important factor after high adsorption capacity in the selection of materials as adsorbents, the examination of adsorption kinetics [pseudo-first-order model (PFO), pseudo-second-order model (PSO), and intraparticle diffusion model (IPD)] is very useful to determine the type of adsorption process and the rate-controlling step.

At the end of the adsorption equilibrium period, approximately 3 mL of solution was taken from the experimental medium and filtered through a Millex-LCR hydrophilic PTFE membrane filter (0.45 μm pore size, 25 mm diameter). The concentrations of Hg(II) remaining in solution were determined by measuring the absorption of the Hg(II)-diphenyl carbazide complex solution. The percentage of Hg(II) removal (*R*, %) and the amount of adsorbed adsorbate (*q*_e_, mg/g) were calculated by Eqs. (1) and (2), respectively:

1 $${\text{R}}(\%) =\frac{{({\text{C}}_{{\text{o}}} - {\text{C}}_{{\text{e}}}) \times {{100}}}}{{\text{Co}}}$$

2 $$\:{\text{qe}}({\text{mg}}/{\text{g}})\: = \:\frac{{({\text{C}}_{{\text{o}}} - \:{\text{C}}_{{\text{e}}} )\: \times \:{\text{V}}\:}}{{\text{m}}}$$

Here, the Hg(II) concentrations in the initial and equilibrium solutions are *C*_o_ (mg/L) and *C*_e_ (mg/L), respectively, the Hg(II) solution volume is *V* (L), and the amount of GVP is *m* (g).

### Adsorption isotherms

Adsorption isotherm investigations are used to determine the maximal theoretical capacity of an adsorbent in relation to a certain adsorbate. It is also possible to clarify details on the adsorption mechanism. Adsorption isotherms correspond to graphs that show how the amount adsorbed on the surface of an adsorbent at a constant temperature is related to the amount not adsorbed. This is achieved by applying equilibrium experimental data to equations, sometimes referred to as adsorption isotherm models, which aid in quantifying the amount of material bound to the surface at a specific liquid concentration. To determine the optimal isotherm model that explains the adsorption equilibrium, model parameters are computed from regression graphs produced by fitting the experimental data to the isotherm equations. Despite the abundance of isotherm models in the literature, it is advised to select isotherm models that most closely reflect the adsorbent material’s chemical and physical characteristics. Langmuir, Freundlich, and Temkin isotherm models are frequently employed. According to the Langmuir model, saturation monolayer adsorption of adsorbate molecules on homogeneous binding sites on the adsorbent surface results in the greatest adsorption capacity. The isothermal viability of Langmuir is estimated using the dimensionless separation factor constant (*R*_L_). According to the Freundlich model, multilayer adsorption of adsorbed molecules on uneven adsorbent surfaces is the cause of the highest theoretical capacity. The Temkin isotherm considers the impact of indirect interactions between the adsorbent and the adsorbate on the adsorption process and assumes that the decrease in the heat of adsorption is linear. Table 1 contains the equations for the models under investigation^3,5^.

**Table 1:** Adsorption isotherm models and equations.

Isotherm models	Linear equations	Parameters	
Langmuir	$\:\frac{{\text{C}}_{\text{e}}}{\text{q}\text{e}}\text{ = }\frac{\text{1}}{{\text{K}}_{\text{L}} \times {\text{q}}_{\text{m}}}\text{ + }\frac{{\text{C}}_{\text{e}}}{{\text{q}}_{\text{m}}}\text{}$	*q*_e_ (mg/g)	Adsorption capacity at equilibrium
		*C*_e_ (mg/L)	Equilibrium concentration of adsorbate
		*q*_m_ (mg/g)	Maximum adsorption capacity
		*K*_L_ (L/mg)	Langmuir constant
	$\:{\text{R}}_{\text{L}}\text{ = }\frac{\text{1}}{\text{1 + (}{\text{K}}_{\text{L}}\times{\text{C}}_{\text{o}}\text{)}}$	*R*_L_	Dimensionless separation factor
		0 < *R*_L_<1	Langmuir assumption, Appropriate
		*R*_L_>1	Langmuir assumption, Unfavourable
		*R*_L_=0	Langmuir assumption, Irreversible
Freundlich	$\:\text{ln}\text{q}\text{e}\text{ = }\text{ln}{\text{K}}_{\text{F}}\text{ + }\frac{\text{1}}{{\text{n}}_{\text{f}}}\text{ln}{\text{C}}_{\text{e}}$	*q*_e_ (mg/g)	The amount of adsorbate adsorbed
		*C*_e_ (mg/L)	Adsorbate concentration in solution
		*K*_F_ ()	Freundlich constant
		1/*n*_*f*_	An empirical parameter indicating surface heterogeneity in the range of 0–1
		*n*_*f*_ < 1	Chemical adsorption
		*n*_*f*_ = 1	The adsorption process is linear
		*n*_*f*_ > 1	Physical adsorption
Temkin	$\:\text{q}\text{e}\text{}\text{ = }{\beta}_{\text{1}}{\text{In}\text{K}}_{\text{T}}\text{ + }{\beta}_{\text{1}}{\text{In}\text{C}}_{\text{e}}$	*q*_e_ (mg/g)	Equilibrium adsorption capacity
		*C*_e_ (mg/L)	Adsorbate equilibrium concentration
		*β*_1_ (J/mol)	Adsorption heat constant
		*K*_T_ (L/mg)	Equilibrium binding constant

### Adsorption thermodynamics

The information required to perform thermodynamic studies is obtained by investigating the dependence of the adsorbent’s adsorption behaviour on a particular adsorbate as a function of temperature. Based on the adsorption data at various temperatures, the parameters needed to assess the thermodynamic nature (Gibbs free energy change (Δ*G*^o^), standard entropy change (Δ*S*^o^), and standard enthalpy change (Δ*H*^o^)) are computed and assessed using the formulas given in Table 2^5^. Table 3 provides a summary of the thermodynamic parameter values and the interpretation of the adsorption nature^31^.

**Table 2:** Van’t Hoff equation and thermodynamic parameters.

Equations		Thermodynamic parameters	
$\Delta {\text{G}}^{ \circ } \:{\text{ = }}\:{\text{ - RT}}\:{\text{lnK}}_{{\text{D}}}$		Δ*G*^o^	Gibbs free energy change
		*K*_D_	Adsorption equilibrium constant
		R (8.314 J/mol.K)	Ideal gas constant
		T (K)	Absolute temperature
$\Delta G^\circ \: = \:\Delta H^\circ - T\Delta S^\circ$		Δ*H*^o^	Enthalpy change
		Δ*S*^o^	Entropy change
$\:\ln {\text{K}}_{{\text{E}}} = - \frac{{\Delta {\text{H}}^{\circ } }}{{R{\text{T}}}}{\text{ }} + {\text{ }}\frac{{\Delta {\text{S}}^{\circ } }}{{\text{R}}}$	Van’t Hoff equation		
$\:\text{ln}\text{}\text{k}\text{ = }\text{ln}\text{}\text{A}\text{ - }\frac{\text{E}\text{a}}{\text{RT}}$	Arrhenius equation	*E*a (kJ/mol)	Activation energy
		A	Arrhenius constant
		*K*	Apparent rate constant

**Table 3:** Thermodynamic parameter values and nature of adsorption.

Thermodynamic parameters	Value	Comment
Δ*H*^o^	+	Endothermic
	−	Exothermic
	< 84 kJ/mol	Physical adsorption
	84–420 kJ/mol	Chemical adsorption
Δ*S*^o^	+	Increase in disorder
	−	Increase in order
Δ*G*^o^	+	Non-spontaneous
	−	Spontaneous
	(− 20) − 0 kJ/mol	Physical adsorption
	(− 80) − (− 400) kJ/mol	Chemical adsorption

### Adsorption kinetics

Kinetic studies are conducted in order to determine the nature of interactions between adsorbate and adsorbent and to comprehend the mechanism underlying the adsorption process. Adsorption kinetics studies how quickly the adsorption process proceeds and, consequently, how long it takes to achieve equilibrium. The slowest step is the rate-controlling step of the overall adsorption rate^3^. Kinetics is the measure of the amount of time that passes between the start of adsorption and equilibrium. Adsorption kinetics can be used to calculate the rate of the adsorption process as well as the effective adsorbate-adsorbent contact time. It is mandatory to examine the kinetics in finding the adsorption rate (and therefore its effectiveness) and the adsorption mechanism.

The most popular models for describing adsorption kinetics are the pseudo-first-order model, pseudo-second-order model, and intraparticle diffusion model. Table 4 provides the linear model equations for the pertinent models. The pseudo-first-order model developed by Lagergren suggests the physical adsorption mechanism, while the pseudo-second-order model, according to Ho and McKay, suggests chemical adsorption. The intraparticle diffusion model was proposed by Weber and Morris to explain the rate change in stratified equilibrium systems^5,32^.

**Table 4:** Equations of the studied kinetic models.

Kinetic model	Linear equations	Kinetic parameters	
Pseudo-first-order	$\:\text{ln(}\text{q}\text{e}\text{}\text{-}\text{q}\text{t}\text{}\text{) = ln}\text{q}\text{e}\text{}\text{ - }{\text{k}}_{\text{1}}\text{t}$	*q*e (mg/g)	Adsorption capacity at equilibrium
		*q*t (mg/g)	Adsorption capacity at time t
		*t* (min)	Time
		*k*_1_ (1/min)	Pseudo-first-order rate constant
Pseudo-second-order	$\:\frac{\text{t}}{\text{q}\text{t}}\text{ = }\frac{\text{1}}{{\text{k}}_{\text{2}}{\text{q}\text{e}}^{\text{2}}}\text{ + }\frac{\text{t}}{\text{q}\text{e}}$	*k*_2_ (g/mg min)	Pseudo-second-order adsorption constant
Intraparticle diffusion	$\:\text{q}\text{t}\text{ = }{\text{k}}_{\text{d}\text{}}{\text{t}}^{\text{1/2}}\text{ + }\text{Q}$	*k*_d_ (mg g min^− 2^)	Diffusion rate constant
		*Q* (mg/g)	Constant related to boundary layer thickness

### Optimization using response surface methodology (RSM)

Changing one factor at a time (OFAT) is the standard approach for researching the factors influencing adsorption. This method ignores the notion that the combination of many elements can either amplify or negate the impact of each other by examining each factor’s influence independently. These days, response surface methodology (RSM) and other statistical and engineering techniques are employed to address this issue. There are fewer experiments needed with this approach. Graphs and suitable models illustrating the linear or quadratic effects of the variables under investigation are produced using this method, which also takes into account the link between different elements. Thus, the effects and interactions of potential effective factors on the desired response through RSM are examined with the least number of specific experiments, making it easier to obtain ideal process conditions. RSM is an effective method to optimize multiple variables to find the optimum response with the minimum number of experiments using a designed series of experiments. Using mathematical and statistical tools, response surface methods like Box-Behnken design (BBD), central composite design (CCD), and Doehlert design (DD) adapt empirical models to the experimental data in relation to the experimental models. Consequently, the variable Xi is coded as xi for the statistical process using the transformation that follows (Eq. 3):

3 $$x_{{\text{i}}} = {\text{ }}\left( {X_{{\text{i}}} - X_{{\text{o}}} } \right)/\Delta x$$

where *x*_i_ is the variable *X*_i_’s dimensionless coded value, *X*_o_ is the variable *X*_i_’s value at the center point, and Δ*x* is the step change. The optimization process is formulated as a quadratic model of the relationship (including linear terms) between the response, factors, and interactions (Eq. 4).

4 $$\:{\text{y}}\:{\text{ = }}\:\beta _{{\text{0}}} \:{\text{ + }}\:\sum {\:_{{{\text{i = 1}}}}^{{\text{k}}} } \beta _{{\text{i}}} {\text{x}}_{{\text{i}}} \:{\text{ + }}\:\sum {\:_{{{\text{i = 1}}}}^{{\text{k}}} } \beta _{{{\text{ii}}}} {\text{x}}_{{\text{1}}}^{{\text{2}}} \:{\text{ + }}\:\sum {\:_{{{\text{1}} \le {\text{i}} \le {\text{j}}}}^{{\text{k}}} } \beta _{{{\text{ij}}\:}} {\text{x}}_{{\text{i}}} {\text{x}}_{{\text{j}}} \:{\text{ + }}\varepsilon \:$$

where *β*₀, *β*_i_, *β*_i__i_, and *β*_ij_ are the regression coefficients as the constant term, linear effect term, quadratic effect term, and interaction effect term, respectively; *x*_i_ and *x*_j_ are the factors; *ε* is the random error; and *y* is the response of the factors. They are estimated using multiple nonlinear regression analyses^27,28^.

CCD provides high-quality and meaningful estimates of linear and quadratic interaction effects of process parameters affecting the process. With *k* being the number of independent components, CCD uses three different kinds of space points: 2^*k*^ factorial, 2*k* axial, and 1 central (*N*_c_) experimental runs. Five replications of the central point were made in order to assess the “experimental error”. Equation (5) establishes the number of experimental runs (*N*) needed for modelling and process optimisation^33^.

5 $$\:\text{N}\hspace{0.17em}=\hspace{0.17em}{2}^{k}\hspace{0.17em}+\hspace{0.17em}2k\hspace{0.17em}+\hspace{0.17em}N\text{c}$$

GVP was employed as a novel adsorbent in this study to remove Hg(II) from aqueous solutions. Utilising the response surface methodology (RSM), which includes central composite design (CDD), the removal conditions were examined. The effects of two independent variables, such as the amount of adsorbent and the concentration of analyte, on the response functions were also examined. The best conditions were identified by optimising the removal percentage (*R*, %)^34^ and adsorption capacity (*q*_e_, mg/g)^35^ for Hg(II). Then, a suitable regression model was proposed for the adsorption system. In order to minimize the uncontrolled factor effects, 13 randomly ordered experimental runs were designed, including 4 factorial points, 4 star points, and 5 central points determined by Eq. (5). Additionally, statistical modelling was done to examine how the removal efficiency and adsorption capacity were affected by experimental independent variables including the amount of GVP and the concentration of Hg(II) ions. Additionally, analysis of variance (ANOVA) was used to assess the significance of the independent variables and the accuracy of the model. According to ANOVA, the significance of factors as well as their interactions was evaluated using the Fisher test (*F*) and its associated probability *P*. The model with the highest *F*-value and the lowest *P*-value indicates a better adjustment of the input parameters^36^. The coefficient of determination (*R*^2^) and adjusted coefficient of determination (*R*^2^_adj_) were used to express how appropriate the polynomial model equations were. Regression analysis, 3*D* response surface, and contour plots were used to estimate the ideal circumstances.

## Results and discussion

### Characterization/structural and morphological study

#### FTIR analysis

**Fig. 1:**
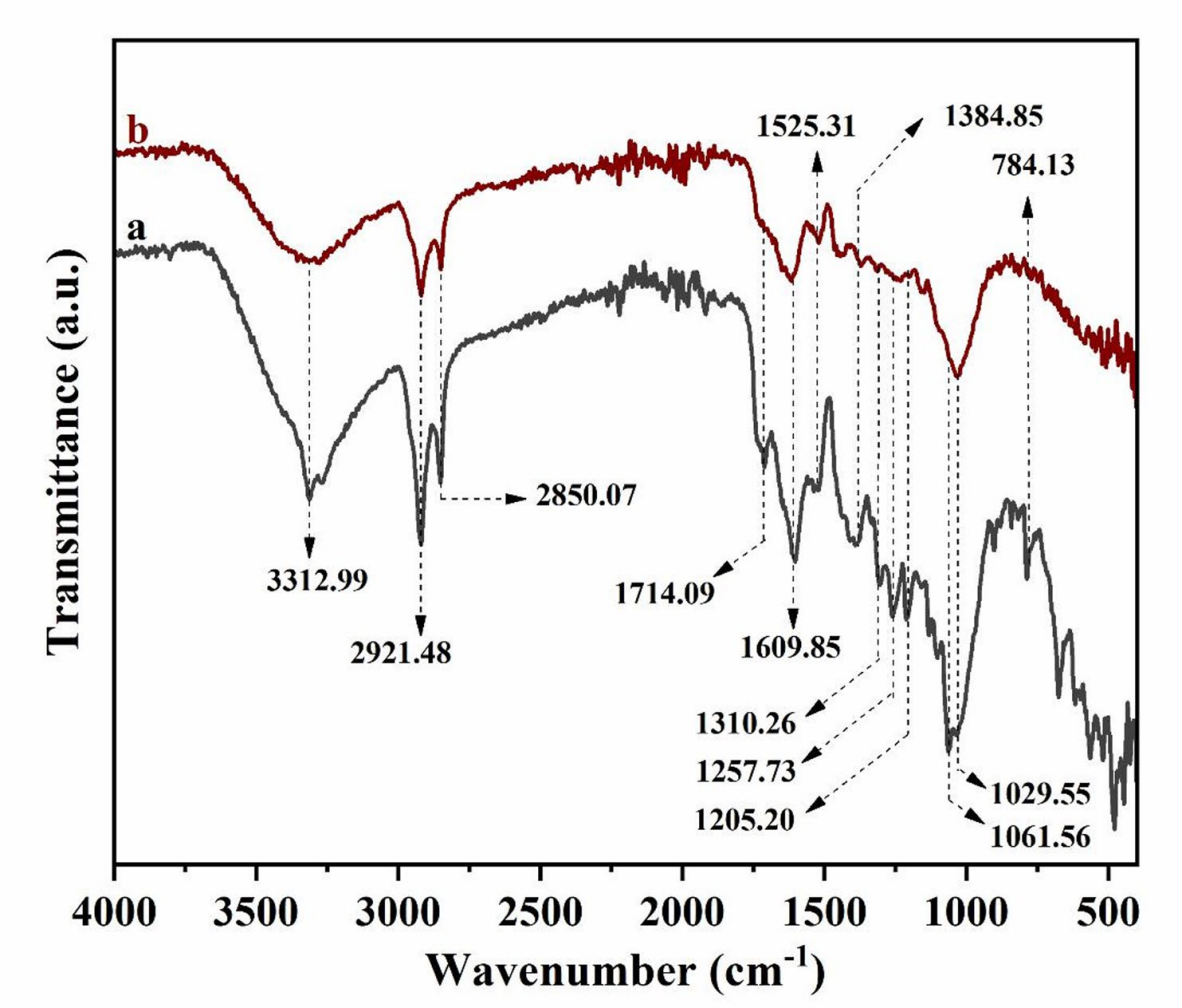
FTIR spectra of GVP before (**a**) and after (**b**) Hg(II) adsorption.

The functional groups of GVP were investigated using Fourier transform infrared spectrophotometry (FTIR), and the morphological properties were investigated using scanning electron microscopy (SEM). FTIR analyses were carried out before and after adsorption to identify both the active functional groups of GVP and the adsorption mechanism by associating these functional groups with Hg(II) during the adsorption of Hg(II) onto GVP. The FTIR spectra of GVP and Hg(II)-loaded GVP obtained under optimum adsorption conditions were examined in the range of 500–4000 cm^−1^ and are presented in Fig. 1. Since GVP is a lignocellulosic material, all the characteristic bands found in cellulose, hemicellulose, and lignin should be seen in the GVP spectrum. To determine the functional groups, the spectrum was compared with those found in the literature. The absorption bands observed in the GVP spectrum confirmed the existence of several functional groups that could aid in adsorption. In Fig. 1a, there are three distinct bands in the GVP spectrum, ranging from 1000 to 1200 cm^−1^, 1500 to 1700 cm^−1^, and 2800 to 3700 cm^−1^. The broad band at 3312.99 cm^−1^ is associated with the –OH stretching vibration associated with cellulose and lignin. The double peak at 2921.48 and 2850.07 cm^−1^ is associated with the symmetric –CH stretching of the alkane group and represents the aliphatic –CH stretching vibration of GVP in the cellulose-hemicellulose structure associated with lignin^37,38^. The peaks at 1714.09 cm^−1^ and 1609.85 cm^−1^ in the band obtained in the range of 1600–1800 cm^−1^ are associated with the C=O stretching vibrations of carboxylic acid groups (–COOH) associated with hemicellulose and their derivatives such as carboxylates. The band gap of 1650–1500 cm^−1^ is –NH bending vibration. The peak at 1384.85 cm^−1^ is aromatic C=C groups. The peak at 1310.26 cm^−1^ is methyl C–H groups. The peak at 1257.73 cm^−1^ is an aromatic ring group. The peaks at 1205.20 cm^−1^ and 1029.55 cm^−1^ are C–O groups of carboxylic acids found in lignin. Lignin in the GVP structure was also confirmed by the strong C–O band at 1061.56 cm^−1^^19^. The peak at 784.13 cm^−1^ may be the C–H bonds in cellulose. When FTIR images are fully evaluated, there is evidence for the presence of hydroxyl, carbonyl, carboxyl, ester, and aromatic groups that are important for adsorption^39,40^. Functional groups that contain oxygen, such carboxyl and hydroxyl, provide an environment that is conducive to Hg(II) adsorption^41^.

The FTIR spectrum (a) of GVP shows more peaks compared to the spectrum (b) of Hg(II)-loaded GVP, and changes in the intensities of some peaks were observed after Hg(II) adsorption. The intensity of the –OH band at 3312.99 cm^−1^ for GVP decreased after Hg(II) adsorption. The peak intensities related to the –CH stretching vibration of the carboxylic group (2921.48 and 2850.07 cm^−1^) and the C=O stretching vibration (1714.09 cm^−1^) decreased. While the intensity of the C=O stretching band at 1609.85 cm^−1^ decreased, the peak intensity of the –NH band at 1525.31 cm^−1^ did not change. The peak of the weak band related to the C–N stretching of –NH_2_ observed for GVP at 1061.56 cm^−1^ disappeared. In general, a decrease in the intensity of all peaks was observed in the FTIR spectrum of GVP after Hg(II) adsorption. The intensities of absorption bands associated with O–H, C=C, and C=O groups decreased after Hg(II) adsorption, indicating that these groups could interact with Hg(II). These spectrum shifts demonstrated that GVP has functional groups that were capable of interacting with Hg(II) and that these functional groups were crucial to the removal of Hg(II). FTIR study supported the idea that GVP might be employed as a possible biosorbent for Hg(II) adsorption and validated the interactions between the adsorbent and the adsorbate.

#### SEM and BET analysis

**Fig. 2:**
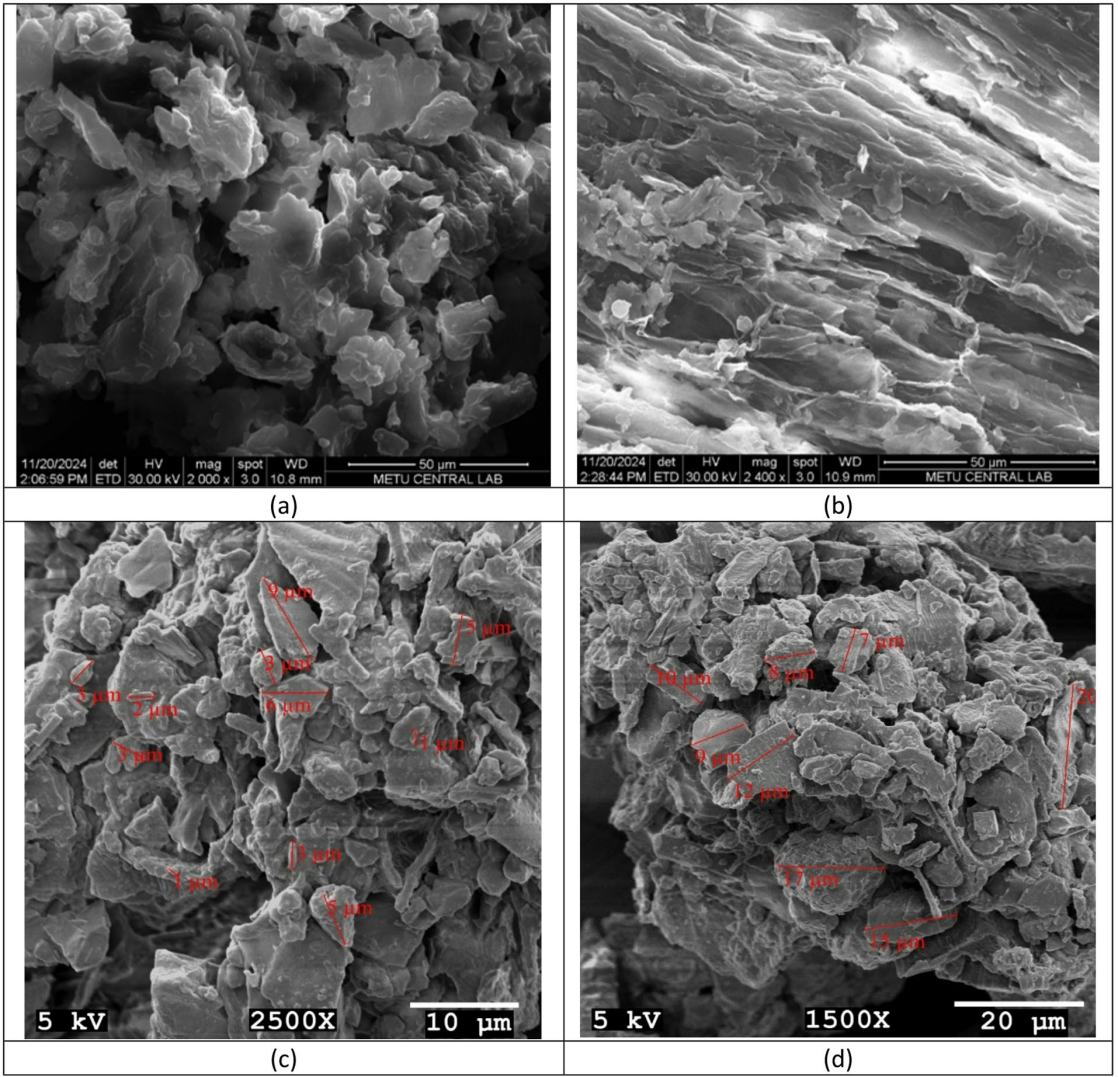
SEM micrographs of GVP showing before (**a**) and after (**b**) Hg(II) adsorption, and the particle size distributions at low (**c**) and high (**d**).

The structural and morphological properties of GVP were evaluated by BET and SEM analyses. SEM examination was done to look for potential changes in GVP’s surface shape before and after adsorption. SEM micrographs provide important information supporting the potential use of GVP as an adsorbent. Large surface area, porous structure, and heterogeneity can increase the adsorption capacity of the adsorbent for different pollutants. However, SEM analysis after Hg(II) adsorption will help to better understand the adsorption mechanism and the effectiveness of the adsorbent. SEM (Scanning Electron Microscopy) micrographs showing the changes in the surface morphology of GVP and Hg(II) adsorption are given in Fig. 2a,b. In Fig. 2a, it is seen that GVP has a complex and heterogeneous structure. In Fig. 2a, pores and spherical particles of various sizes were observed in the GVP structure, which would allow Hg(II) ions to penetrate into the cellulosic structure and interact with surface groups. The differences in particle sizes may be due to the different components of the GVP (shell, core, pulp, etc.). Surface roughness and porous structure can create active sites for surface events such as adsorption. These morphological properties of the adsorbent are important for adsorption. This supports the usability of GVP as an adsorbent in wastewater treatment. Adsorption efficiency is increased because the adsorbent’s wide surface area creates more active binding sites, or a bigger contact area between the adsorbent and the adsorbate. The surface morphological change was evident in SEM micrographs of the GVP taken both before and after adsorption. Figure 2b is the SEM image of GVP after Hg(II) adsorption. It can be seen that the complex and heterogeneous structure of GVP changed after Hg(II) adsorption. After adsorption, the GVP surface presented a surface morphology in which small agglomerates formed due to the interaction with Hg(II) ions were dispersed. These new morphologies formed on the GVP surface confirmed that the sorption mechanism occurred during the removal of Hg(II) from aqueous solutions. Although there were still fibrous structures and voids on the surface, some significant differences were observed. These differences reflect the changes caused by Hg(II) ions on the structure of GVP. Changes such as less roughness and a smoother appearance on the surface, slight changes in the shape and size of the particles, and a decrease in the number and size of the pores suggest that Hg(II) ions were successfully adsorbed onto the GVP surface and that GVP was an effective adsorbent for Hg(II) adsorption. This clearly showed that the presence of Hg(II) ions on the adsorbent promoted the removal of Hg(II) ions.

The SEM images in Fig. 2c,d shows that the average particle diameters of GVP was characterized by the clustering of spherical particles, along with sharp rod- and plate-type particles, onto large bulky particles The average particle diameters of GVP were measured between approximately 1 μm and 20 μm.

The BET surface area, total pore volume and average pore size for GVP were determined using BET analysis. The surface area of ​​GVP was determined as 2.983 m^2^/g by multipoint BET analysis, and the cumulative adsorption surface area was determined as 6.988 m^2^/g by Barrett-Joyner-Halenda (BJH) analysis. The total pore volume and average pore size of the GVP surface were determined as 0.023 cm^3^/g and 4.435 nm, respectively. These results suggest that the GVP has a mesoporous structure, which provides suitable textural properties for adsorption applications.

#### TGA-DTA analysis

**Fig. 3:**
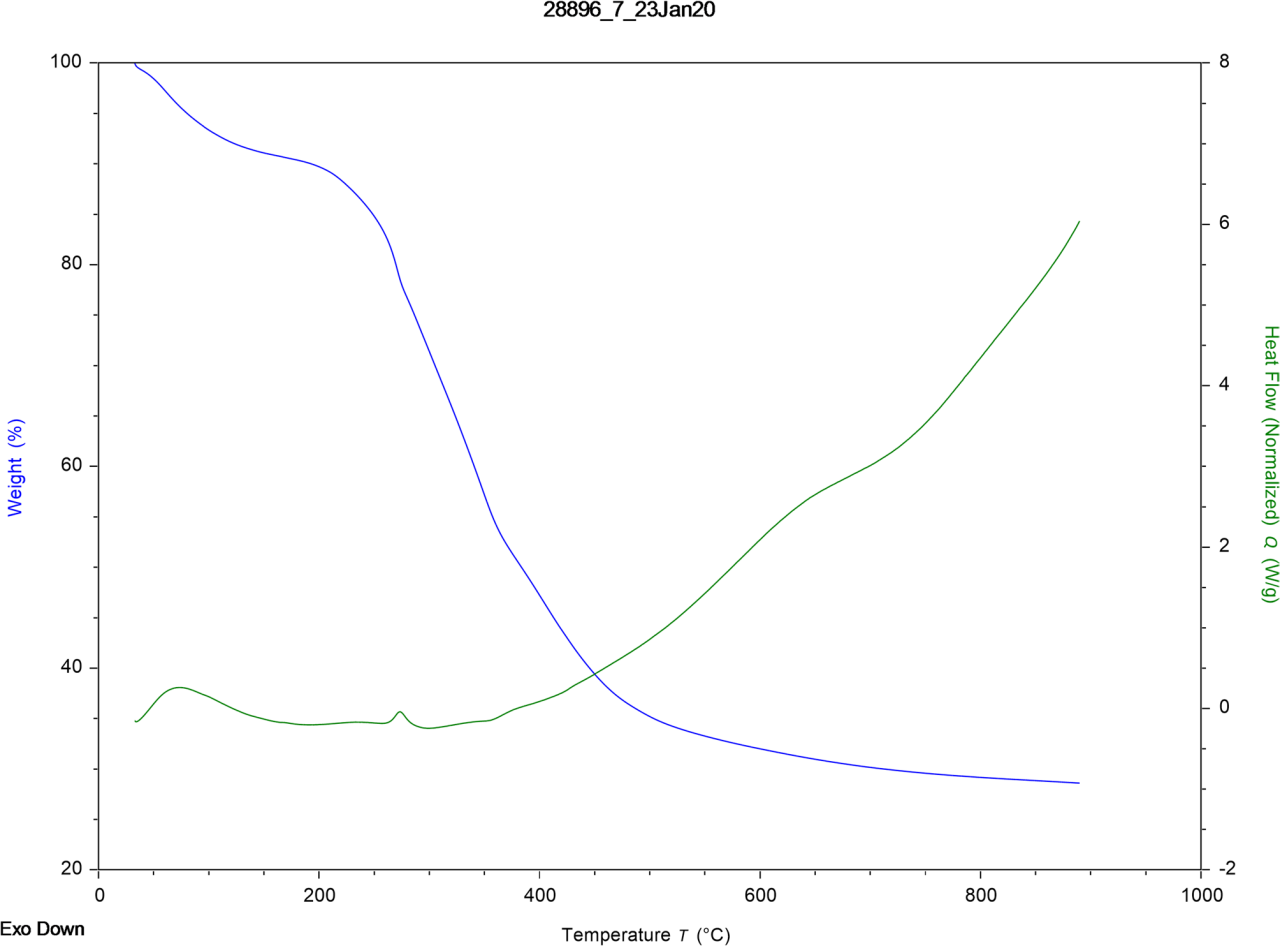
TGA-DTA analysis results for GVP (Blue line: TGA curve, Green line: DTA curve).

Thermogravimetric Analysis (TGA) and Differential Thermal Analysis (DTA) curves are powerful tools used to study a sample’s thermal characteristics. These techniques are used to study the physical and chemical properties of a material that change with temperature. By interpreting the curves, information can be obtained about the sample’s thermal degradation mechanism, composition, thermal stability and phase transition. Figure 3 shows the TGA and DTA curves for GVP. The TGA curve shows that the mass of the sample decreases as the temperature increases. This mass loss may be due to the evaporation of volatile components (water, organic acids, etc.) in GVP, thermal decomposition, and gas evolution as a result of the reaction. The mass loss stages with different slopes in the TGA curve indicate that different components of GVP decompose or change thermally at different temperatures. The constant mass at high temperatures indicates the ash content or carbonized residue of the sample. Thermogravimetric analysis contributes to the characterization of the thermal stability of cellulosic materials, as it is possible to lead to chemical and structural changes. That is, this study uses the decomposition of the material’s constituent components to reveal information on the material’s thermal stability^42^.

Examining the TGA and DTA curves together provides more comprehensive information about the thermal behaviour of GVP. Endothermic (downward) and exothermic (upward) peaks can be seen in the DTA curve. For example, mass loss in the TGA curve and endothermic peaks in the DTA curve may correspond to the same thermal event, such as evaporation of water or degradation of organic components. Exothermic peaks in the DTA curve, together with mass loss in the TGA curve, may indicate the presence of events such as combustion or oxidation. TGA-DTA curves for GVP provided information about the thermal stability, composition, and thermal degradation mechanism of GVP. The peaks and mass loss stages in the curves may reflect the thermal behaviours of different components in GVP (cellulose, hemicellulose, lignin, pectin, organic acids, etc.). In the TGA-DTA curves in Fig. 3, continuous mass loss was observed in the sample in three different mass loss stages with increasing temperature. In general, the first stage corresponds to moisture loss and decomposition of very light volatile compounds with a temperature range below 160 °C. The decomposition of hemicellulose, cellulose, and lignin, referred to as the second stage, takes place between 209 and 395 °C. Around 395–571 °C is when the complex and thermally stable structure is burned, or charified, in the final step of lignocellulose biomass^43^. Hemicellulose decomposes at 220–315 °C, cellulose at 315–400 °C, and lignin at 160–900 °C^39^. At low temperatures (< 500 °C), mass loss involves endothermic reactions, while at high temperatures (> 500 °C), it involves exothermic reactions^40^. TGA-DTA curves for GVP showed similar characteristics in the decomposition temperature ranges reported in the literature for lignocellulose materials. In particular, the effect of the heating temperature of GVP on the Hg(II) adsorption ability can be explained by examining the results of TGA analysis. Based on Fig. 3, it is clear that the initial mass loss occurs below 160 °C. GVP showed mass loss between approximately 30 and 120 °C, mainly due to the removal of water molecules from the adsorbent and the resulting opening of pores on its surface. Therefore, the optimum adsorption capacity is achieved in this temperature range. A more significant decrease in the mass of GVP was observed in the temperature range from 250 to 430 °C, which is attributed to the decomposition of organic compounds. In the next stage, the decomposition of GVP into highly volatile and low molecular weight carbon products may result in a decrease in its mass^44^.

#### Point of zero charge (pH_*pzc*_)

**Fig. 4:**
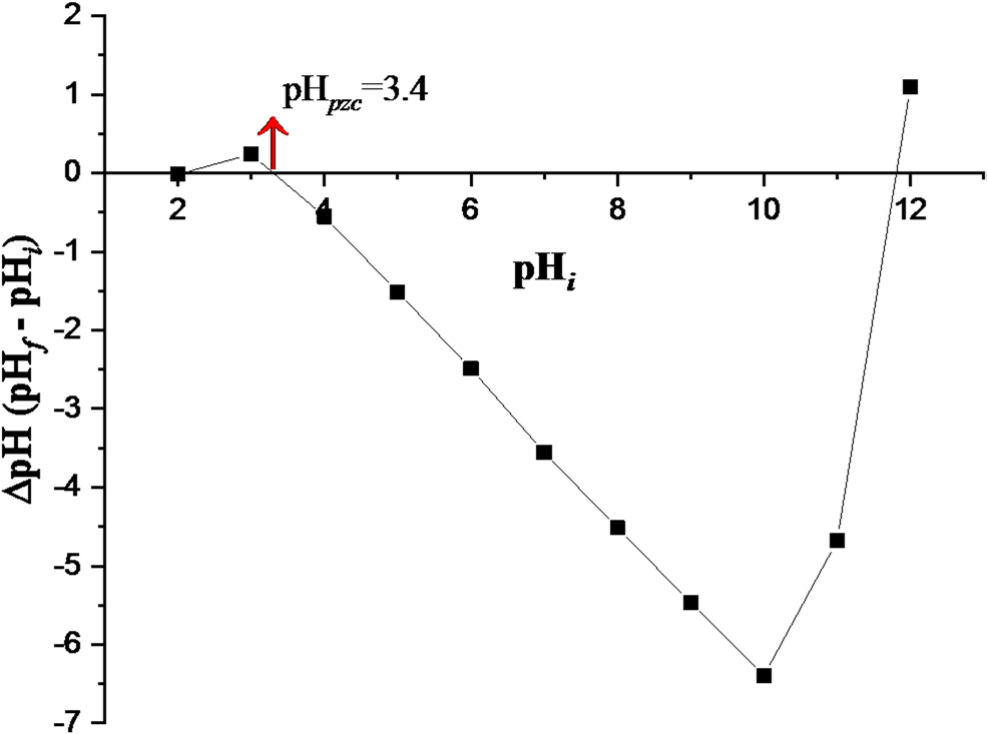
pH at the point of zero charge (pH_*pzc*_).

For the determination of pH_*pzc*_, 0.250 g of adsorbent was added to 50 mL amber bottles containing 25 mL of 0.3 M NaCl solution for crude GVP. First, the pH of the solutions in the bottles was adjusted to different initial pH values (pH_*i*_= 2–12) using 0.1 M NaOH or 0.1 M HCl, and the suspensions were shaken on an orbital shaker for a period of 48 h until the equilibrium pH was reached. Then, the final pH value (pH_*f*_) of each solution was measured. The pH changes at equilibrium (ΔpH = pH_*f*_ – pH_*i*_) was calculated. Finally, the material’s pH_*pzc*_ was determined by plotting the ΔpH graph against the pH_*i*_. The point where ΔpH and pH_*i*_ overlapped was recorded as the pH_pzc_ value^41^. The pH_*pzc*_ values for GVP were determined as 3.4 from the curve given in Fig. 4. At this pH_*pzc*_ value, the surface charge density of the GVP adsorbent is zero. That is, it indicates that the adsorbent has neither a positive nor a negative charge at this pH. Below these values, the adsorbent surface has a net positive charge, and above it, it has a net negative charge. In other words, the surface of the material will be negatively charged when pH > pH_*pzc*_ and positively charged when pH < pH_*pzc*_^45^. The increasing pH level causes an increase in the concentration of OH^−^ ions on the adsorbent’s surface, which promotes the adsorption of cations through electrostatic interactions. On the other hand, when the pH value is lower than pH_pzc_, an increase in positive charges causes electrostatic repulsion between ions of the same species, which leads to a decrease in the adsorption ability. Normally, the adsorption of anions is favoured at a solution pH lower than pH_*pzc*_, while the adsorption of cations is promising at a solution pH greater than pH_*pzc*_ (Fito et al., 2023). Since the adsorbent’s surface density is negatively charged, it is anticipated that cationic Hg(II) will be adequately absorbed. Consequently, for GVP, the adsorption of Hg(II) is preferred at solution pH levels higher than 3.4.

#### Effect of solution pH

The ability of pH to guide the interactions between the adsorbent and the adsorbate makes it a crucial parameter for adsorbate removal. The pH of the solution generally has an impact on the adsorbate charge, the functional groups of the adsorbent, and the surface charge in the solution. The pH depends on the adsorbent and adsorbate; there is no set guideline. In some cases, especially for heavy metals, pH increases the removal rate. For example, it can be said that higher pH is beneficial in terms of adsorption capacity, that is, it improves the adsorption capacity by causing deprotonation of hydroxyls on the surface thanks to electrostatic interactions between the adsorbent and the adsorbate. At low pH, it can be said that the repulsive force can restrict the access of adsorbate ions to the adsorption sites. It has also been stated that the metal can precipitate as hydroxide in the alkaline condition depending on the pH. All of these findings demonstrate how intricately pH affects how contaminants are adsorbed by adsorbents^46^. In the case of Hg(II) ion, which is investigated as adsorbate in this study, when pH < 6, water molecules surround Hg(II) and form, was observed at pH 4^3^. In this study, it was found that solution pH was not very effective in achieving maximum removal efficiency in the pH range examined.

6 $${\text{Hg}}^{{{\text{2}} + }} + {\text{ H}}_{{\text{2}}} {\text{O}} \rightleftharpoons {\text{HgOH}}^{ + } + {\text{ H}}^{ + } \quad {\text{K}}_{{\text{1}}} = {\text{ 1}}0^{{ - {\text{3}}.{\text{4}}}}$$

7 $${\text{HgOH}}^{ + } + {\text{ H}}_{{\text{2}}} {\text{O}}~ \rightleftharpoons ~~~{\text{Hg}}\left( {{\text{OH}}} \right)_{{\text{2}}} + {\text{ H}}^{{ + ~}} \quad {\text{K}}_{{\text{2}}} = {\text{ 1}}0^{{ - {\text{2}}.{\text{7}}}}$$

8 $${\text{Hg}}^{{{\text{2}} + }} + {\text{ 2H}}_{{\text{2}}} {\text{O}}~~~~ \rightleftharpoons ~~~{\text{Hg}}\left( {{\text{OH}}} \right)_{{\text{2}}} + {\text{ 2H}}^{ + } \quad {\text{K}}_{{{\text{total}}}} = {\text{ 1}}0^{{ - {\text{6}}.{\text{1}}}}$$

**Fig. 5:**
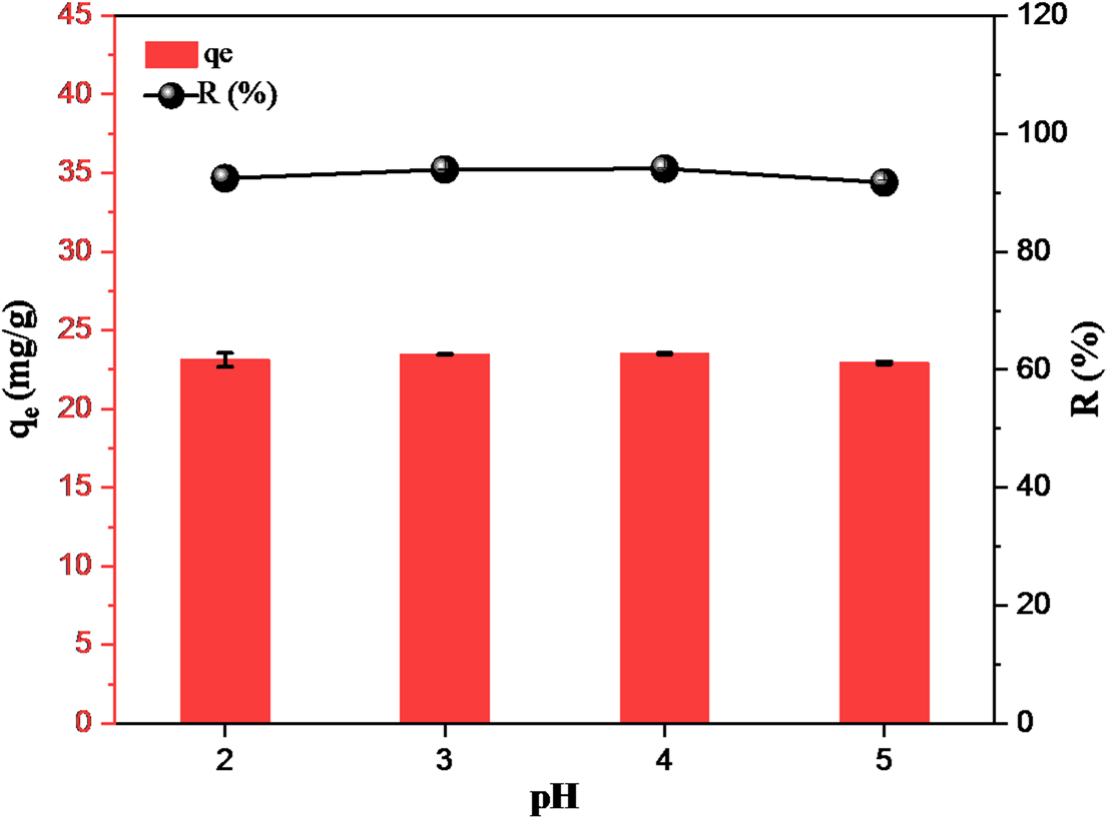
Effect of pH change on Hg(II) removal (C_Hg_: 100 mg/L, m: 4.0 g/L, t: 30 min).

The pH effect was carried out to evaluate the adsorption removal efficiency and capacity of GVP towards Hg(II) ions and ensure that the adsorbent functions effectively for environmental applications. The pH of the solution can significantly affect the electrostatic interactions between the adsorbent surface and the adsorbate. When the pH of the solution is greater than pH_pzc_ of the GVP (pH > pH_pzc_), the surface of the GVP exhibits a negatively charged state, which makes the adsorbate attractive for adsorbing positively charged ions. When the pH of the solution is less than pH_pzc_ (pH < pH_pzc_), the hydroxyl groups of the GVP are protonated, so the surface of the GVP behaves as if it were positively charged. As the pH of the solution increases, the positive surface charges of the GVP decrease, resulting in a decrease in the repulsion between the positive charges of the adsorbent and the adsorbate. On the other hand, as the pH of the solution decreases, the electrostatic forces between the increasing positive adsorbent charges and the negative charges of the adsorbate increase^47^. In the studied pH range (2–5), no significant change was observed in the removal percentage and capacity of GVP (Fig. 5). Therefore, Hg(II) removal by GVP can be directly carried out without any adjustment when the pH of the solution medium is in the range of 2–5. Under these conditions, approximately 94*%* Hg(II) removal efficiency and 24 mg/g removal capacity were obtained.

#### Effect of initial Hg(II) concentration

**Fig. 6:**
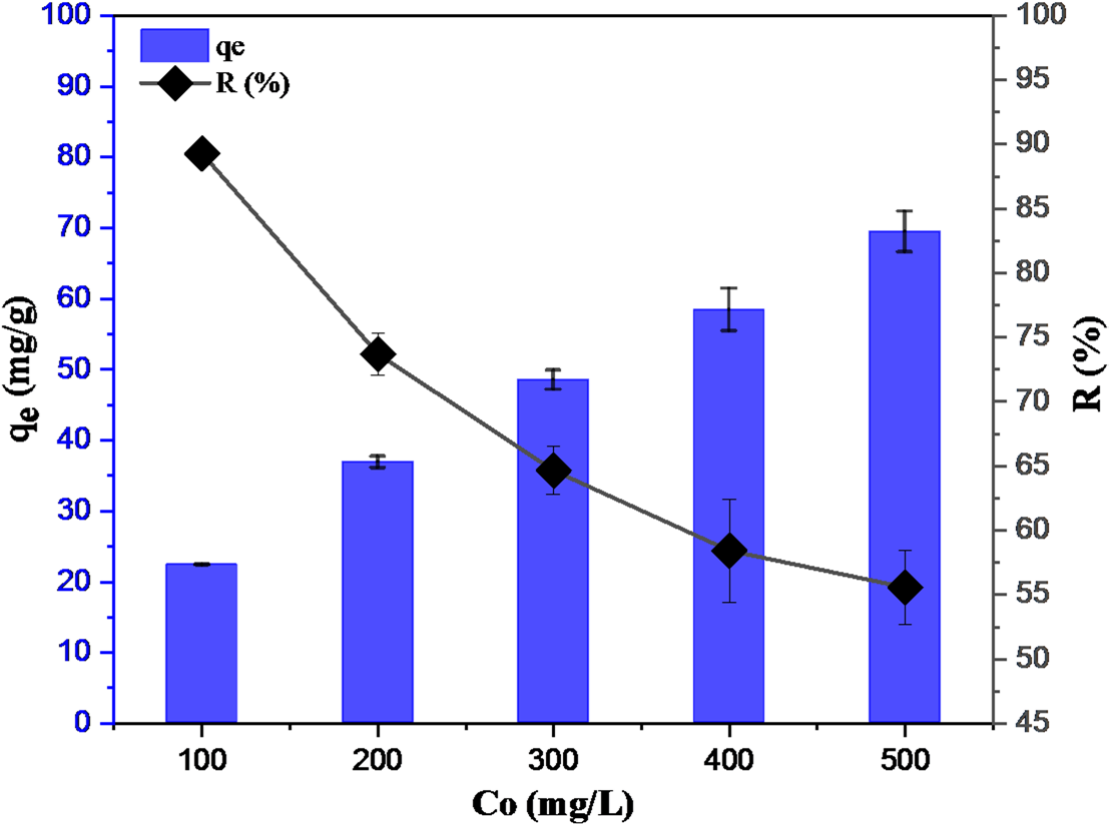
Effect of initial Hg(II) concentration on removal (pH: ~4, m: 4.0 g/L, t: 30 min).

Another crucial factor is the initial concentration of the adsorbate. Generally speaking, the quantity of empty binding sites on the adsorbent surface is directly proportional to the concentration of adsorbate. The excess number of adsorbates compared to the number of available adsorption sites or the rise in cohesive forces between molecules in the solution could be the cause of the decrease in removal rate as the initial adsorbate concentration rises. The *R* % and *q*_e_ plots for GVP as a function of initial Hg(II) concentration are displayed in Fig. 6. Intuitively, *q*_e_ increases with increasing *C*_o_, while *R* % decreases for all adsorbents. The plot clearly shows that the optimum concentration for 70*%* Hg(II) removal for GVP is 100 mg/L. Because with increasing initial concentration, the driving force for mass transfer also increases and the adsorption amount of Hg(II) increases^48^. Although Hg(II) binds to the active sites at low concentrations through attractive forces like electrostatic and van der Waals, the interaction between the adsorbent and the adsorbed solution is hampered by the mass transfer resistance between the aqueous and solid phases. Higher concentrations allow more Hg (II) to adsorb on the adsorbent because it can overcome the resistance. That is, it can be said that the increase in collisions between Hg(II) ions and adsorbent particles enables an increase in the adsorption capacity (mg/g) as the initial concentration of the adsorbate increases.

#### Effect of adsorbent amount

**Fig. 7:**
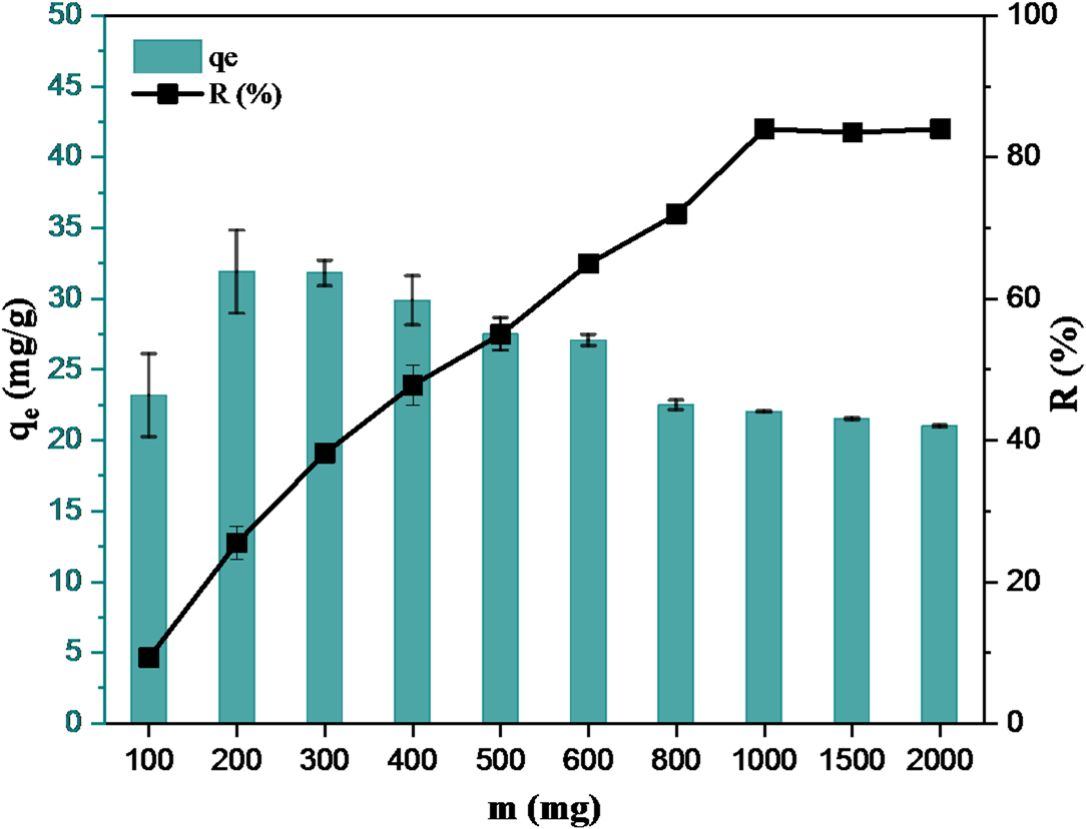
Effect of adsorbent amount on Hg(II) removal (pH: ~4, C_Hg_: 100 mg/L, t: 30 min).

Only the adsorbent amount changed in adsorption. Thus, as expected, it was observed that the amount of Hg(II) adsorbed per gram of adsorbent decreased with increasing adsorbent amount at constant Hg(II) concentrations (Fig. 7). This decrease may be due to the increase in unused areas on the adsorbent surface in adsorption as the amount of adsorbent increases. This situation can be shown by increasing the adsorbate concentration. The amount of Hg(II) adsorbed and the percentage of Hg(II) removal were calculated against the increase in amount of adsorbent (Fig. 7) and it was found that the adsorbent between 100 and 2000 mg showed maximum adsorption at constant Hg(II) concentration. A higher amount of adsorbent generally permits a larger number of available unoccupied sites at a fixed adsorbate concentration, which raises the removal rate and lowers the adsorption capacity. However, eventually this increase reaches a plateau after a certain adsorbent dose. Indeed, the number of sites adsorbing the adsorbate is greater than the number of adsorbate, so an equilibrium is reached. Therefore, it is necessary to determine the optimum amount of adsorbent to avoid unnecessary amounts and waste^46^.

#### Effect of contact time and kinetics

**Fig. 8:**
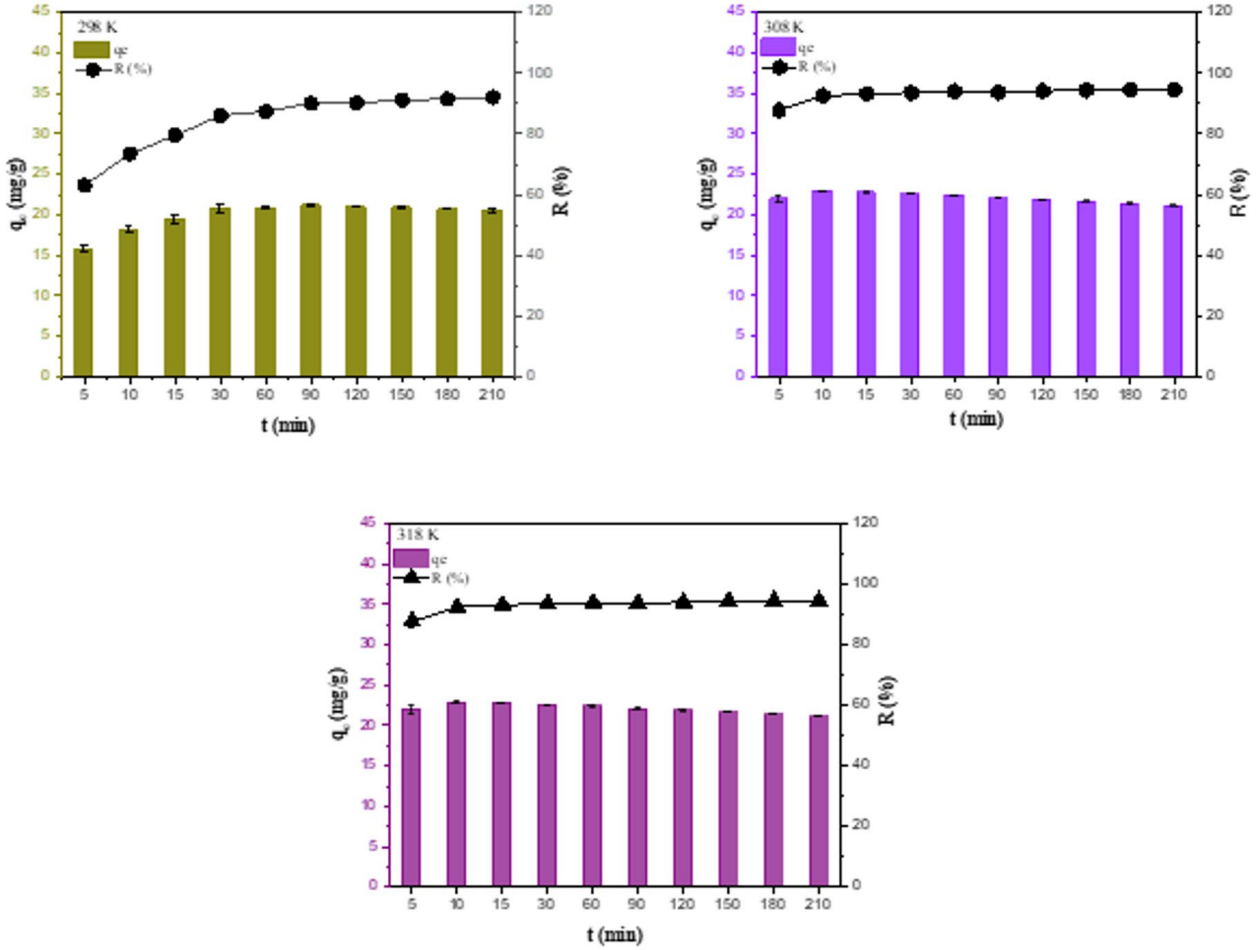
Effect of contact time on Hg(II) removal at 298, 308 and 318 K (pH: ~4, C_Hg_: 100 mg/L, *m*: 0.100 g).

Particularly when researching the adsorbent efficiency, the contact time is one of the most crucial variables. For the adsorbate concentrations between the adsorbent surface and the aqueous solution to reach equilibrium, it specifies the average contact time needed. The adsorption mechanisms can be ascertained by conducting kinetic research on the adsorption of the adsorbate. Before achieving equilibrium, the adsorption rate usually rises quickly at first because of the large surface area. Repulsion between the adsorbed molecules and those remaining in the solution, particularly for ionic species, and the reduction of unoccupied sites are what cause the equilibrium state^46^. Adsorption experiments were conducted using a solution temperature of 25 °C, a pH ~ 4, a Hg(II) concentration of 100 mg/L, and an adsorbent amount of 4.0 g/L to examine the influence of time. The removal efficiencies and capacities of GVP with time were shown in Fig. 8. Since there were many vacant active sites at the initial stage, the adsorbent showed a rapid Hg(II) removal within the first 30 min. Then, the removal slowed down and remained almost constant. The adsorption capabilities remained unchanged after 30 min, as seen in Fig. 8. It was established that 30 min was the equilibrium adsorption time for GVP. This period was the ideal time for the other studies.

**Fig. 9:**
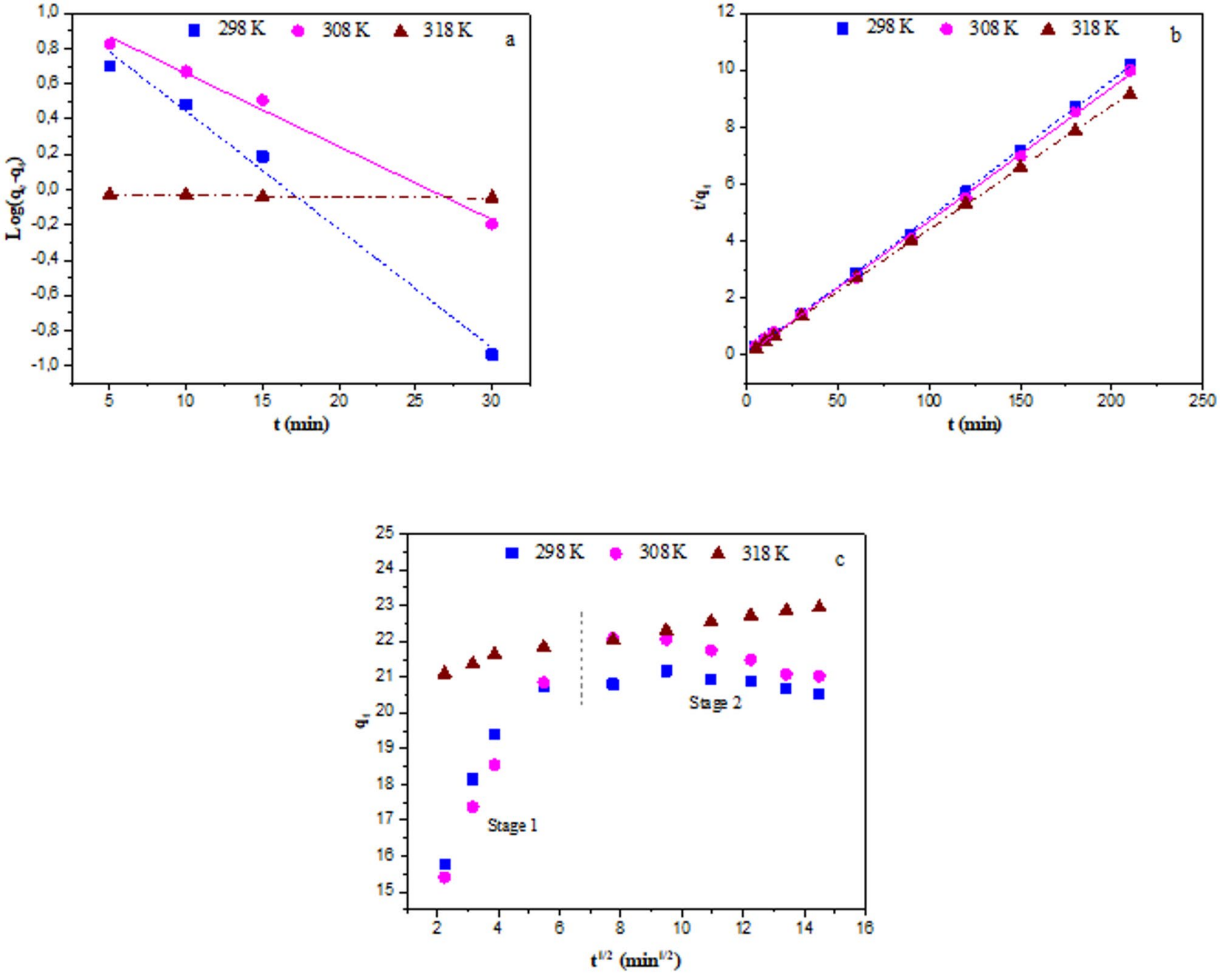
For Hg(II) adsorption by GVP, plots of the pseudo-first-order kinetic model (**a**), pseudo-second-order kinetic model (**b**), and intraparticle diffusion model (**c**) obtained at various temperatures (pH: ~4, *C*_Hg_: 100 mg/L, m: 4.0 g/L).

The process of adsorption is physicochemical. Kinetic studies are essential for an effective adsorption process, which involves the mass transfer of a solution to a solid surface. Because they offer details on the adsorption mechanism, these investigations are favoured. Mass transfer of solution to a solid surface is an adsorption process, and kinetic studies are necessary for an efficient adsorption process. These studies are preferred because they provide information about the adsorption mechanism. Kinetic studies are conducted to assess the rate-determining stage in surface adsorption, where the physicochemical interaction between the adsorbent and the adsorbate occurs, and to ascertain how quickly the adsorption process proceeds. In this study, it was performed to explain the mechanism of the adsorption process and to reveal the nature of adsorbate/adsorbent interactions. Since a high adsorption rate is an important factor in the selection of materials as adsorbents after high adsorption capacity, the adsorption kinetics study is very useful to determine the type of adsorption process and the rate-controlling step^49^. The most commonly used pseudo-first order, pseudo-second order and intraparticle diffusion kinetic models were also investigated for the adsorption of Hg(II) onto GVP. The rate and interaction between Hg(II) and GVP were evaluated using the linear equations given in Table 3 for these three kinetic models. The constants and *R*^2^ values ​​of these models were obtained from the graphs shown in Fig. 9 and are presented in Table 5. Keeping the amount of adsorbent, the concentration of Hg(II), and the volume of solution constant, adsorption was monitored for 200 min. Aliquots (100 µL) of the solution were extracted at predetermined intervals, and the concentrations of Hg(II) were measured. The slope of the log(*q*_e_–*q*_t_) plot against *t* was used to determine the pseudo-first-order rate constant (*k*_1_), and the intercept was used to determine the equilibrium adsorption capacity (*q*_e_). From the intercept and slope of the *t*/*q*_t_ plot against *t*, the pseudo-second-order rate constant *k*_2_ and the equilibrium adsorption capacity (*q*_e_) values were determined, respectively. In the intraparticle diffusion model, *Q*, which is a constant related to the boundary layer thickness, and the diffusion rate constant *k*_D_ were determined from the intercept and slope, respectively, by plotting *q*_t_ against *t*_1/2_. The linear fit results and the corresponding parameters obtained from the kinetic analyses are summarized in Table 5. From the linear models, it is clearly seen that the *R*^2^ value of the pseudo-second order model is relatively close to the pseudo-first order model. In this case, it is not possible to prefer one model over the other, but the adsorption capacity (*q*_e_cal) calculated at time *t* of the pseudo-second-order model exhibited a value closer to the experimental adsorption capacity (*q*_e_exp). But compared to the pseudo-first-order model, the pseudo-second-order model, which states that the adsorption rate is proportional to the square of the number of free active sites, was noticeably more accurate. Since the experimental equilibrium adsorption capacity (*q*_e_) value was found to be 20.76 mg/g, the pseudo-second-order model calculated this value to be 20.79 mg/g, but the pseudo-first-order model calculated it to be 13.02 mg/g. This was valid for all temperatures. Therefore, this led to the conclusion that the pseudo-second-order model was more suitable to explain the kinetic process. The *R*² values ​​for the intraparticle diffusion model were very small compared to other models, indicating that this model was not suitable to describe the adsorption kinetics. These findings suggest that chemisorption may be the mechanism underlying GVP’s adsorption of Hg(III)^50,51^.

**Table 5:** Kinetic modelling parameters.

T (K)	qe_exp_ (mg/g)	Pseudo-first-order			Pseudo-second-order				Intraparticle diffusion		
		k_1_ (1/min)	qe_cal_ (mg/g)	*R* ^2^	k_2_ (g/mg.min)	qe_cal_ (mg/g)	h (mg/g.min)	*R* ^2^	k_d_ (mg/g.min^2^)	Q (mg/g)	*R* ^2^
298	20.76	0.1543	13.02	0.9906	0.1112	20.79	48.08	0.9997	0.275	17.62	0.5101
308	20.85	0.0953	11.84	0.9909	0.0948	21.37	43.29	0.9991	0.384	16.97	0.5695
318	22.95	0.0017	0.94	0.9486	0.0305	22.98	16.11	0.9996	0.140	20.95	0.9825

#### Thermodynamic studies

Thermodynamic analysis is another aspect required to identify and comprehend the nature of adsorption. Gibbs free energy change (ΔG^o^), enthalpy change (ΔH^o^), and entropy change (ΔS^o^) are the thermodynamic descriptions studied for the adsorption effect of Hg(II) on GVP. These three parameters explain the adsorption process’s energy changes and fundamental mechanism. To study the thermodynamics, the adsorption process was carried out at different temperatures (298 K, 308 K and 318 K). The thermodynamic parameters of the adsorption process were obtained using the adsorption data at 298, 308 and 318 K and are given in Table 6. To determine the impact of temperature, the vant Hoff equation was employed. The study’s negative ΔG^o^ value suggests that the adsorption of Hg(II) onto GVP is both feasible and spontaneous. Furthermore, the ΔG^o^ values ((− 20)–0 kJ/mol) suggest that the adsorption of Hg(II) onto GVP may be influenced by physical forces such van der Waals, hydrogen bonding, and dipole-dipole interactions^52^. The ΔH^o^ value (31.20 kJ/mol) was positive, indicating that the adsorption of Hg(II) on GVP was an endothermically driven physical (< 84 kJ/mol) process. The ΔS^o^ value was found to be 109 J/K.mol, suggesting randomness at the adsorbent/solution interface. It indicated the endothermic nature of the adsorption process for Hg(II) by GVP. Activation energy, *E*_a_ value is an important factor indicating the rate at which the adsorption rate changes with temperature. The larger the *E*_a_ value, the slower the reaction will occur. According to the pseudo-second-order kinetic model for Hg(II) adsorption onto GVP, the *E*_a_ value was found to be 9.58 kJ/mol. The positive *E*_a_ value confirmed that higher temperatures favoured the removal of Hg(II) onto GVP, and the process was endothermic in nature^53^. Also, the magnitude of the *E*_a_ value can give an idea about the type of sorption. *E*_a_ value less than 40 kJ/mol supports physical adsorption^54^. In summary, as described in Table 6, negative values of ΔGº showed a spontaneous and thermodynamically favourable removal process at all temperatures examined. An endothermic and stochastic process for Hg(II) removal was suggested by the positive values of ΔHº and ΔSº^51^.

**Table 6:** Thermodynamic parameters.

T (K)	K_D_	ΔHº (kJ/mol)	ΔSº (J/K.mol)	ΔGº (kJ/mol)	E_a_ (kJ/mol)
298	1.65	31.20	109.00	− 1.41	9.58
308	3.06			− 2.52	
318	3.63			− 3.60	

#### Isotherms studies

**Fig. 10:**
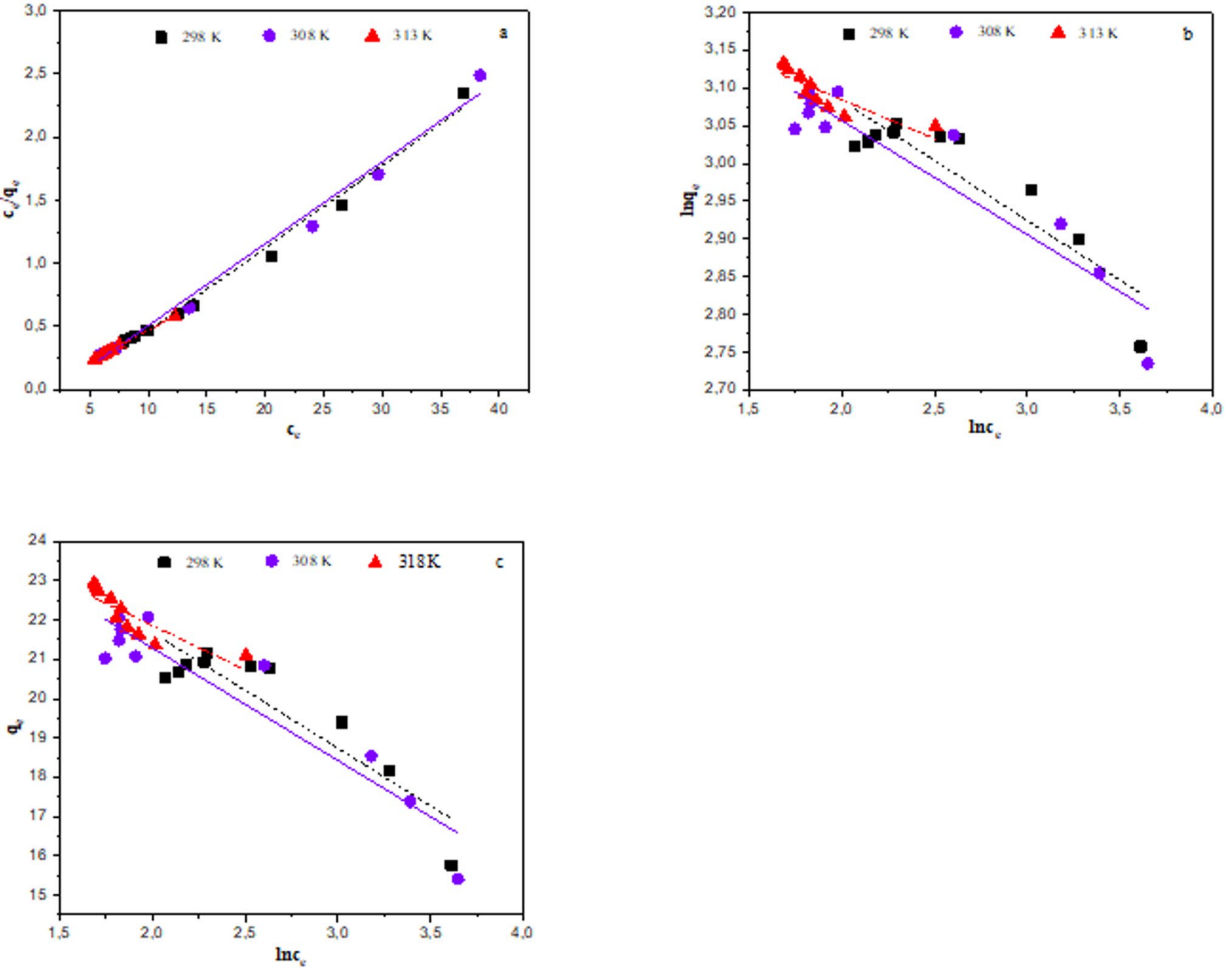
Linear curves of (**a**) Langmuir, (**b**) Freundlich and (**c**) Temkin isotherm equations obtained at different temperatures for Hg(II) adsorption by GVP.

To comprehend how adsorbate molecules disperse between the aqueous and solid phases under equilibrium conditions, adsorption isotherms are utilised. When adsorption and desorption occur at the same rate, an adsorption isotherm provides the equilibrium relationship to characterise the interaction between the adsorbent and adsorbate at a constant temperature. In order to find the model that best fits the experimental data, equilibrium isotherm modelling was carried out by applying the equilibrium data collected at different temperatures (298–318 K) to the isotherm models (e.g., Langmuir, Freundlich, and Temkin isotherms). This modelling provides ideas about the capacity, surface properties, and adsorption mechanism of the adsorbent^49^. Using linear forms of the Freundlich, Temkin, and Langmuir isotherms, the adsorption behaviour of Hg(II) on GVP was investigated. A multilayer adsorption on heterogeneous surfaces is described by the Freundlich isotherm, whereas the Langmuir isotherm presumes that the adsorbate molecules form a monolayer with a homogeneous surface on the adsorbent^55^. To take into consideration the impact of indirect adsorbent-adsorbate interactions on adsorption, a Temkin isotherm was also created. Additionally, this isotherm makes the assumption that each molecule in the layer experiences a linear drop in adsorption temperature as a result of the interactions^56^. Graphical representations of Langmuir, Freundlich and Temkin isotherms as linear curves of Hg(II) adsorption by GVP are presented in Fig. 10. Table 7 shows the calculated adsorption parameters. Accordingly, Langmuir, Freundlich, and Temkin isotherms have coefficients of determination *R*^2^ 0.9890, 0.8082, and 0.8217, respectively. The calculated data support the experimental data with relatively high *R*^2^ values ​​ (*R*^2^ > 0.99) compared to other models, in which the Langmuir model adsorbs Hg(II) onto GVP in a monolayer manner, suggesting the homogeneity of the adsorption sites on the adsorbent. The adsorption process of Hg(II) takes place in the active site of the monolayer surface of the adsorbent, resulting in a homogenous surface. The adsorption equilibrium constant is denoted by the Langmuir constant. In the case of an adsorbent with a high Langmuir constant, the most active sites of the adsorbent are used, and a significant decrease in the residual concentration of the adsorbate can be achieved. As a result, water treatment uses a small amount of adsorbent^4^. The maximum adsorption capacity (*q*_max_) of Hg(II) adsorption onto GVP was found to be 20.76 mg/g. In addition, the calculated *q*_max_ and *K*_L_ values ​​of the Langmuir isotherm were 15.24 mg/g and − 0.35 L/mg, respectively. In addition to showing the adsorption capacity, the Langmuir-type isotherm model’s *K*_L_ value also conveys the adsorbent’s affinity for the adsorbate. In this case, high *K*_L_ values imply strong binding. Additionally, less free energy is needed when the *K*_L_ value is higher. Adsorbent and adsorbate have a weak binding, as indicated by the *K*_L_ value of − 0.35 L/mg in this study. This provides insight into the adsorption process’s reversibility and physical adsorption mechanism. On the other hand, the Langmuir isothermal feasibility (*R*_L_) related to the density was determined as −0.03, suggesting that the adsorption process is favourable. In parallel, the fit value of the Langmuir model is supported by the regression coefficient and the calculated isotherm parameter values ​​with an *R*^2^ value of 0.99, indicating that it is sufficient and acceptable to explain the equilibrium adsorption data of Hg(II) onto GVP. The Freundlich and Temkin models were not suitable to explain the adsorption of Hg(II) onto GVP due to the fact that the *R*^2^ values ​​were not high enough (Table 7). The calculated *q*_max_ values ​​show that adsorption occurs more easily at high temperatures and increases with temperature.

**Table 7:** Isotherm model parameters obtained at different temperatures.

T (K)	q_max.exp_ (mg/g)	Langmuir isotherm				Freundlich isotherm			Temkin isotherm			
		q_max.cal_ (mg/g)	K_L_ (L/mg)	*R*_L_	*R* ^2^	*n*_f_	K_F_ (mg/g)	*R* ^2^	K_T_ (L/g)	β_1_	b_T_ (kJ/mol)	*R* ^2^
298	20.76	15.24	− 0.35	− 0.03	0.9890	− 6.32	29.94	0.8082	0.09	− 2.94	− 827.31	0.8217
308	20.85	15.41	− 0.46	− 0.02	0.9904	− 6.61	28.77	0.8732	0.08	− 2.86	− 850.41	0.8861
318	22.95	19.88	− 1.55	− 0.01	0.9935	− 9.70	26.85	0.7369	0.01	− 2.26	− 1077.25	0.7290

### Adsorption mechanism

**Fig. 11:**
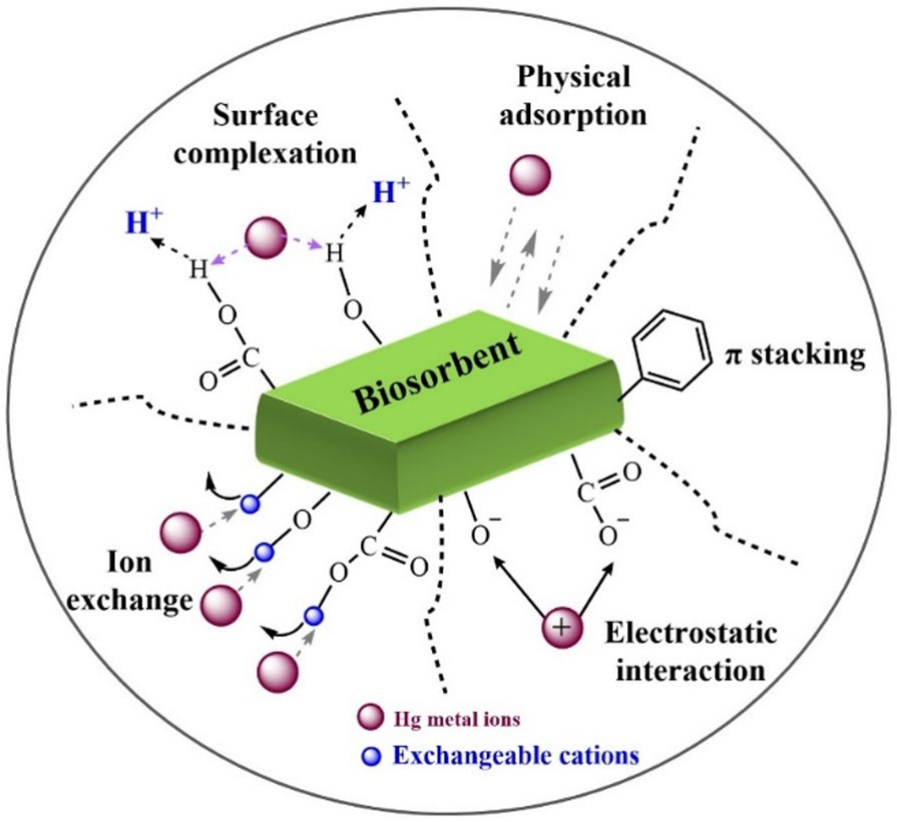
Possible Hg(II) adsorption mechanisms.

The main components of agricultural residues are cellulose, hemicellulose, and lignin, all of which are abundant in different functional groups. Through a variety of interacting processes, these components facilitate the adsorption of Hg(II). Analysing the thermodynamic parameters and the adsorbent characterisation data is crucial to clarifying the reaction processes that may be involved in the adsorption of Hg(II) on GVP. There are hydroxyl, carboxyl, and benzene rings in cellulose, hemicellulose, and lignin. These groups were discovered using FTIR characterisation, and because of their distinct interactions with Hg(II), Hg(II) is integrated onto the surface of GVP. Furthermore, after Hg(II) adsorption, a shift in the intensities of some distinctive peaks (–O–H groups, C=C, C–H, and –C–O bonds) was noted, demonstrating the role of carboxy and phenolic groups in the Hg(II) binding process on the adsorbent surfaces. Furthermore, the analysis of thermodynamic characteristics demonstrated that Hg(II) adsorption on GVP is a physical adsorption. This phenomena is primarily based on n–π interactions, hydrogen bond formation, and electrostatic interactions^57^. Because adsorption studies were conducted at a pH of the solution higher than pH_pzc_, a negative charge developed on the adsorption sites on the adsorbent surface. In order to help Hg(II) ions bind to the adsorbent surface, this negative charge encourages electrostatic contact with them. When the pH effect was investigated, it was shown that the pH of the Hg(II) solution did not have a great effect on the Hg(II) removal in the investigated range. Additionally, Hg(II) and oxygen-containing functional groups (–OH or –COOH) on the adsorbent’s surface (functioning as hydrogen donors) can form a hydrogen bond. The FTIR spectra of GVP and GVP with Hg(II) support this interaction. The creation of hydrogen bonds is shown by the decrease in the –OH stretching vibration strength around 3400 cm^−1^ in these spectra. In conclusion, the π interaction between the benzene rings in GVP and Hg(II) may constitute one of the interactions. Another interaction is the net positive charge of Hg(II) and may lead to electrostatic attraction with negative groups on GVP. The hydrogen bond between GVP and Hg(II) may be another interaction. A final interaction could be the cation exchange between Hg(II) and GVP. Because of this, adsorbents with high concentrations of carboxyl, hydroxyl, and benzene groups are probably better adsorbents. Figure 11 illustrates some possible adsorption techniques.

Generally recognised through the use of the adsorption isotherm, the adsorption mechanism describes the distribution of adsorbed molecules on the adsorbent interface. Predicting the adsorption mechanism is challenging due to the large influence of the contaminants’ types, which include dissociated ions, neutral molecules, polar and non-polar, hydrophobic and hydrophilic. However, the adsorption mechanisms can be explained by the hydrophobic effect, π–π electron donor-acceptor, covalent bond, coulomb interaction, H-bond, π-interaction, surface complexation, electrostatic contact, ion exchange, and dipole interaction. Hydrophobic interactions, hydrogen bonds, and van der Waals forces all demonstrate that the adsorption of Hg(II) was a homogenous, monolayer, and physical process^41^.

### Optimization approach to adsorption process

RSM design of experiments (DoE) methodology was used to investigate the effect of important process parameters (adsorbent amount and initial adsorbate concentration) on the adsorption process and to develop a mathematical model for the prediction of adsorption potential of adsorbent. The optimum conditions for the adsorption of Hg(II) onto GVP were determined using central composite design (CCD).

Two variables were examined in optimisation studies: the initial Hg(II) concentration (Co) and the amount of adsorbent (*m*). For 60 min, 25 mL Hg(II) solutions (pH ~ 4) were stirred at 200 rpm in order to conduct the experiments. The software’s output data included the main effects, factor interactions, coefficients, coefficient standard deviation, and other statistical indices of the models and statistical plots that were produced. 

Since the amount of adsorbent (GVP) and the initial Hg(II) concentration has a significant impact on the adsorption process, these effects were studied. The CDD design, including 13 experiments including two independent variables (Hg(II) concentration and GVP amount) used in this study, and the values ​​of the factors at coded and real levels are presented in Table 8 together with the Hg(II) removal efficiency (*R* %) and removal capacity (*q*_e_) results.

**Table 8:** CCD matrix and experimental results for Hg(II) adsorption onto GVP.

Factors	Symbol	Factor levels				
		–α (–1.414)	–1	0	+ 1	+α (+ 1.414)
*C*_o_ (mg/L)	*X*_1_	100	159	300	441	500
*m* (g)	*X*_2_	0.0100	0.0232	0.0550	0.0868	0.1000

Run	Coded level		Actual level		Results*	
	X_1_	X_2_	C_o_ (mg/L)	m (g)	*R*_mean_% ± S	qe_mean_ ± S
1	− 1	− 1	159	0,0232	50.3 ± 1.0	86.2 ± 1.7
2	1	− 1	441	0,0232	24.5 ± 2.7	116.3 ± 12.9
3	− 1	1	159	0,0868	91.2 ± 0.4	41.8 ± 0.2
4	1	1	441	0,0868	47.7 ± 3.8	60.6 ± 4.8
5	− 1.414	0	100	0,0550	84.8 ± 1.7	38.6 ± 0.8
6	1.414	0	500	0,0550	49.0 ± 2.2	111.3 ± 5.0
7	0	− 1.414	300	0,0100	23.1 ± 1.4	173.4 ± 10.2
8	0	1.414	300	0,1000	71.0 ± 3.0	53.3 ± 2.2
9–13	0	0	300	0,0550	48.7 ± 1.7	65.9 ± 1.7

*(*N* = 2).

Analysis of variance (ANOVA), a statistical approach obtained from experimental data, was used to examine the validity and significance of the parameters in order to optimize the Hg(II) adsorption process. Table 9 shows the ANOVA results. *P*-values were used to assess each parameter’s impact on the model. Table 9 displays, at a 95*%* confidence level, the effects, regression coefficients, standard errors, *T*-, and *P*-values for *R %* and *q*_e_. The model’s relevance is indicated by the ANOVA findings, which show that the *P*-value is less than 0.05. Both the initial concentration of Hg(II) (*C*_o_) and the amount of adsorbent (*m*) variables had significant influence on *R %* and *q*_e_ at the 5*%* significance level (*P* < 0.05), according to the ANOVA results. Given that the *P*-value was less than 0.05, the *C*_o_ and *m* variables had a substantial impact on the *R %* and *q*_e_ values. The interactions of the factors on the adsorption processes were also studied. The parameters with *P*-values ​​greater than 0.05 were eliminated, and model reduction was performed. The proposed models were quadratic. The regression models of *R* % and *q*_e_ in coded units are given in Table 9. In these models, when the effect of a factor is positive, changing the value of a factor (m or *C*_o_) from low to high level (e.g., *m* for *R* % and *C*_o_ for *q*_e_) increases *R* % and *q*_e_. However, if the factor effects are negative, changing the lower level to a higher level (e.g., *C*_o_ for *R* % and *m* for *q*_e_) causes a decrease in both removal efficiency and removal capacity. While the concentration of the initial Hg(II) solution (*C*_o_) positively affects *q*_e_, the amount of adsorbent (*m*) negatively affects *q*_e_. For *R* %, the effects of *m* and *C*_o_ are reversed. Square interaction effects were also found to be effective parameters except for m*m for (*R* % and *C*_o_*C_o_ and *C*_o_*m for *q*_e_). The existence of interaction means that the factors can not only affect the response factor independently but also interact with each other. The interaction effects may be larger or smaller than expected for the direct addition of the factors. The system’s univariate optimisation is unable to identify these synergistic effects^58^. The response surface and contour plots also showed these effects. Standard deviation, coefficient of determination (*R*^2^), difference between adjusted and estimated *R*^2^ are used as model fit parameters for the response values. They should be < 10%, > 0.8, and < 0.2, respectively^59^. *P*-value, *R*^2^, adjusted and estimated *R*^2^ values ​​were used to check the significance of the model. From the ANOVA analysis, the adequacy of the reduced models for GVP was also determined by the coefficients of determination close to 1 (*R*^2^ = 0.9956, *R*^2^adj = 0.9948, and *R*^2^pred = 0.9936 for *R* % and *R*^2^ = 0.9978, *R*^2^(adj) = 0.9975, and *R*^2^(pred) = 0.9968 for *q*_e_). This indicated that just about 1*%* of the overall variation was not explicable by the model. The correlation between the actual and predicted responses for the coded models was about 1. *R*^2^(adj) and *R*^2^(pred) values greater than 0.99 also confirmed the accuracy of the model. That is, a low *P*-value of < 0.05 and *R*^2^(adj) and *R*^2^(pred) values > 0.8 indicated that the model was significant and appropriate. Furthermore, all data points are within ± 10*%* of the predicted and experimental values, which is in statistical agreement. The results revealed that quadratic models with coefficient of determination (*R*^2^) above 0.95 (*P* < 0.05) had good accuracy and good agreement between predicted and experimental data.

**Table 9:** ANOVA results for Hg(II) removal by GVP according to (a) *R* (%) and (b) *q*_e_ (mg/g).

Terms	(a) *R* (%)					
	DF	Adj SS	Adj MS	F-value	*P*-value	VIF
Model	5	9150.64	1830.13	150.28	0.000	
Linear	2	7949.54	3974.77	326.40	0.000	
*C*_o_	1	3601.65	3601.65	295.76	0.000	1.00
*m*	1	4347.89	4347.89	357.04	0.000	1.00
Square interaction	2	1045.25	522.63	42.92	0.000	
*C*_o_*C_o_	1	937.18	937.18	76.96	0.000	1.02
m*m	1	39.86	39.86	3.27	**0.085**	1.02
2-Way interaction	1	155.85	155.85	12.80	0.002	
*C*_o_*m	1	155.85	155.85	12.80	0.002	1,00
Error	20	243.55	12.18			
Lack-of-fit	3	140.73	46.91	7,76	0.002	
Pure error	17	102.83	6.05			
Total	25	9394.19				
Model Summary : *S* = 3.49 *R*^*2*^ = 97.41*% R*^2^(adj) = 96.76*% R*^2^(pred) = 95.09*%*						
Reduced Model Summary: *S* = 1.38 *R*^*2*^ = 99.56*% R*^2^(adj) = 99.48*% R*^2^(pred) = 99.36*%*						
Reduced Regression Equation in Coded Units: *R* (*%*) = 47.508–15.003 *C*_o_ + 16.485 *m* + 8.428 *C*_o_*C_o_ – 4.414 *C*_o_*m						

(b) qe (mg/g)							
Terms		DF	Adj SS	Adj MS	*F*-value	*P*-value	VIF
Model		5	29274.6	5854.9	32.32	0.000	
Linear		2	23983.4	11991.7	66.20	0.000	
	*C*_o_	1	5757.0	5757.0	31.78	0.000	1.00
	*m*	1	18226.3	18226.3	100.62	0.000	1.00
Square Interaction		2	5228.0	2614.0	14.43	0,000	
	*C*_o_*C_*o*_	1	0.0	0.0	0.00	**0.998**	1.02
	m*m	1	5139.7	5139.7	28.37	0.000	1.02
2-Way Interaction		1	63,3	63.3	0.35	0.561	
*C*_o_*m		1	63.3	63.3	0.35	**0.561**	1.00
Error		20	3622.8	181.1			
Lack-of-Fit		3	3221.2	1073.7	45.45	0.000	
Pure Error		17	401.6	23.6			
Total		25	32897.4				
Model Summary : *S* = 13.46 *R*^2^ = 88.99*% R*^2^(adj) = 86.23*% R*^2^(pred) = 76.90%							
Reduced Model Summary: *S* = 1.70 *R*^*2*^ = 99.78*% R*^2^(adj) = 99.75*% R*^2^(*pred*) = 99.68*%*							
Reduced Regression Equation in Coded Units: *q*_e_ (mg/g) = 65.939 + 18.969 *C*_o_ – 33.751 *m* + 19.219 m*m							

DF, Total degrees of freedom; *F*-value, Test statistics; *P*-value, Possibility; Adj SS, Adjusted sum of squares; Adj MS, Adjusted mean squares; VIF, Variance inflation factor; S, Standard deviation; *R*^2^, Coefficient of determination, *R*^2^(adj), Adjusted coefficient of determination; *R*^2^(pred), Predicted coefficient of determination.

**Fig. 12:**
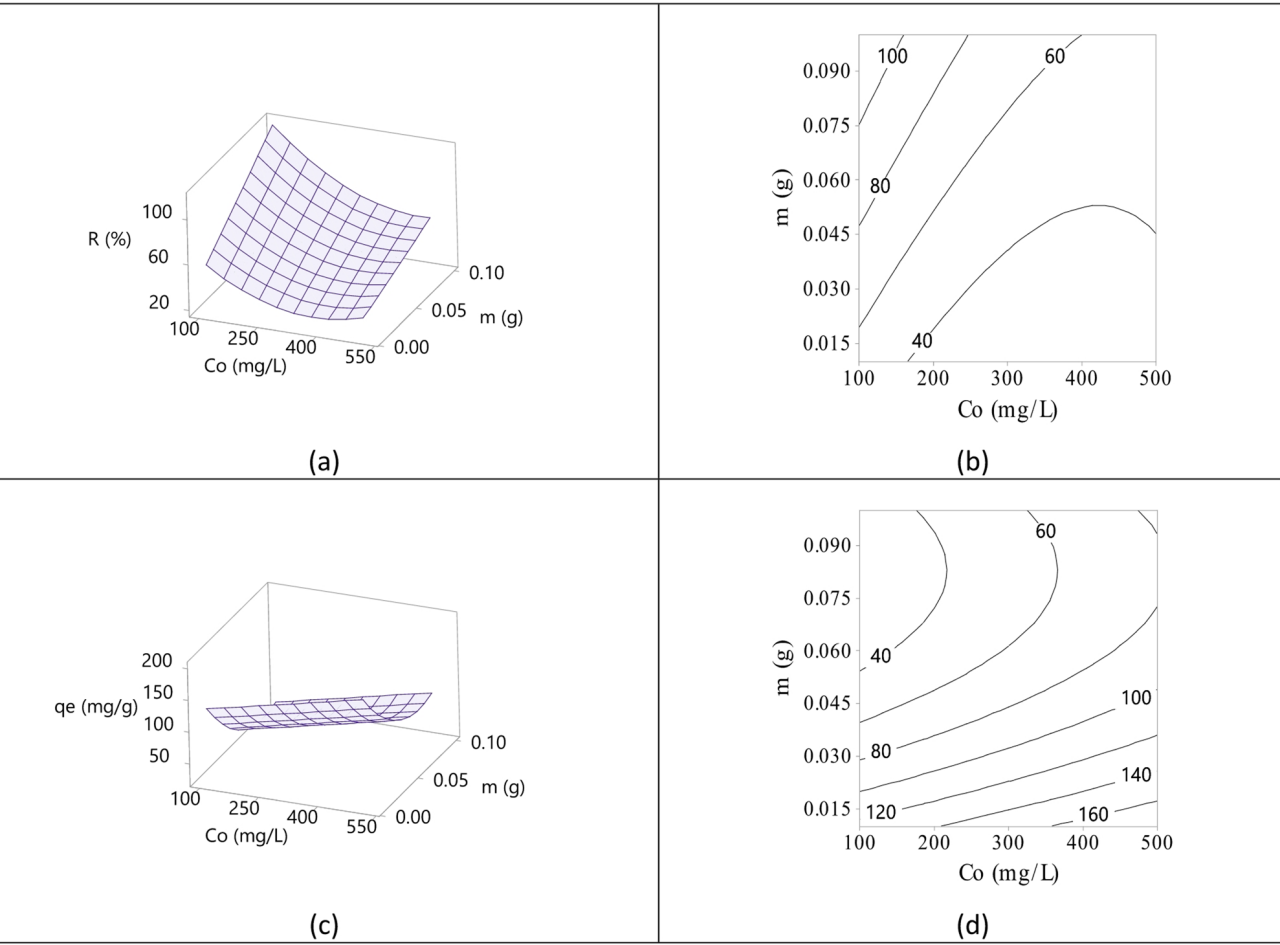
Response surface plots (**a**,**c**) and contour plots (**b**,**d**) for Hg(II) removal percentage and removal capacity with GVP.

RSM uses 3D response surface and contour plots to visualise the variables’ interactions and demonstrate the relationship between the response and effective factors. The experimental results could be easily analysed with the help of 3D interaction (response surface) plots and contour plots of the factors. Figure 12a–d shows the responses of *R* % and *q*_e_ against any of the factors *m* and *C*_o_. These plots show the main and interaction effects of the factors on the adsorption of Hg(II) onto GVP. It was observed that the amount of adsorbent (*m*) was more effective on the removal capacity (*q*_e_) during the adsorption process, while the initial Hg(II) concentration (*C*_o_) was more effective on the removal efficiency (*R* %). The rise in the number of accessible adsorption sites that stayed unsaturated throughout the removal process is responsible for this effect. The presence of interaction indicates that the variables that potentially influence the response factor (*R* % and *q*_e_) do so in an interactive manner rather than independently. *R* % and *q*_e_ may be larger or smaller than expected for the direct effects with the addition of the combined effects. A system optimisation that is univariate is unable to identify these synergistic effects^58^. Figure 11b,d shows an interaction between the factors (*C*_o_ and *m*) via the non-parallel lines in the contour plots for *R* % and *q*_e_. However, two-way interactions were found to be more effective (*P* < 0.05) for *R* % and less effective (*P* > 0.05) for *q*_e_ (Table 9).

The quadratic equation obtained from the model is represented graphically via response surface and contour plots. To estimate the Hg(II) adsorption efficiency on the independent variables, the response surface and contour plots are shown in Fig. 12 (a and b), respectively. The figures show the effects of GVP amount and initial Hg(II) concentration on the adsorption efficiency. As can be seen from the response surface plot, increasing *C*_o_, which has a linear negative effect, decreases *R* %, while increasing *m* constantly increases it. However, due to the *C*_o_^2^ term in the equation, the effect is not fully linear and exhibits a curvilinear behaviour. *C*_o_*m interaction between *C*_o_ and *m* is negative. This causes *R* % to decrease under conditions where both factors are simultaneously high. Maximum *R* % values ​​were obtained at low *C*_o_ and high *m* conditions, while minimum values ​​were observed at high *C*_o_ and low *m* conditions. Contour plot represents different combinations of two experimental variables with the other variable held at different levels. The contour plot summarizes these trends in two dimensions through iso-yield curves and allows estimation of the expected *R* % for different *C*_o_–*m* combinations. These results demonstrate that the optimum conditions of the system are achieved at low initial concentrations and sufficient adsorbent amounts. That is, adsorption efficiency increases with decreasing Hg(II) concentration and increasing GVP amount. The positive increase in adsorption efficiency is attributed to the increase in available surface area and adsorption sites due to the increase in adsorbent amount. For the *q*_e_ (Fig. 12c,d, as *C*_o_ increases, the adsorption capacity *q*_e_ increases almost linearly. The effect of *m* is weak in this range, decreasing *q*_e_ only slightly. The quadratic term for *m* is positive (convex surface). *q*_e_ tends to increase as *m* increases to the limiting values ​​on either side of this axis. Increasing *q*_e_ to the upper limit of *C*_o_ is always beneficial (linear and positive effect). There is a minimum for *m* at the interior point. Therefore, the highest *q*_e_ is generally seen at the lower or upper limits of the design range (and at high *C*_o_). The contour plot clearly shows that *q*_e_ depends most on *C*_o_, while an increase in *m* has only a small decreasing effect in this range.

Derringer’s desirability function (*D*) is a parameter derived from the design program that represents the closeness of a response to its ideal value. Using this function, the optimum values ​​of GVP and m and *C*_o_ for *R* % were determined as 0.090 g and 150 mg/L, respectively, and for *q*_e_ as 0.015 g and 400 mg/L, respectively. Under these conditions, the removal capacity and removal percentage with their standard deviations were 152.2 ± 1.7 mg/g and 96.2 ± 1.4*%*, respectively. To validate the model, experiments were carried out in three replicates under optimum conditions. The experimentally obtained mean response removal percentage (96.9 ± 0.5*%*) and removal capacity (157.3 ± 5.2 mg/g) were within the recommended range, indicating that the model was valid. Under conditions where 96.2% removal efficiency was obtained, the adsorption capacity of GVP was found to be 32.20 mg/g from the model.

The adsorption capacity obtained in the present study was compared with the values reported in the literature for various biomass-based adsorbents in Table 10. GVP showed an adsorption capacity comparable to most other biosorbents, highlighting its potential as an effective adsorbent for Hg(II) removal from aqueous solutions.

**Table 10:** Comparison of Hg(II) adsorpsiyon capacities of various bioadsorbents.

Bioadsorbent	Adsorption capacity (mg/g)	References
Marine algae (*Ulva lactuca Linnaeus*, 1753)	125.6	^60^
Malt spent rootlets particles	50.4	^61^
Candlenut shell particles	37.18	^1^
Grape vinegar pomace	32.20	This study
Coffee waste particles	31.75	^18^
Bamboo leaf powder	27.11	^16^
Ulva sp.	0.377	^62^
Water hyacinth Banana peels	0.0898 0.0919	^13^

## Conclusion

The purpose of this work was to assess the possibility of employing grape vinegar pomace, an agricultural waste, to remove Hg(II) from aqueous solutions. Both univariate and multivariate approaches were used to examine how various parameters affected the adsorption process. To describe the rate control steps and explain the adsorption mechanism, kinetic, thermodynamic, and equilibrium isotherm models were examined. The Langmuir model was followed by the adsorption isotherm. The adsorption kinetics of Hg(II) on GVP were best explained by the pseudo-second-order model, which also demonstrated a significant correlation (*R*^2^ > 0.99). The thermodynamic analysis revealed that the adsorption process was both spontaneous (ΔGº < 0) and endothermic (ΔHº > 0). It was primarily physical in character, controlled by n-π interactions, hydrogen bonds, and electrostatic attraction. The adsorbent’s affinity for the adsorbate was demonstrated by the entropy values (ΔS° > 0). Furthermore, the CCD-RSM optimisation approach successfully optimised the effective adsorption parameters, and the experimental and predicted values showed excellent agreement. The RSM approach with CCD exhibited a very good performance in predicting Hg(II) adsorption. Moreover, this modelling (*m*: 0.090 g and *C*_o_: 150 mg/L) showed that GVP had an adsorption capacity of 32.20 mg/g when the optimum Hg(II) removal efficiency was 96.2*%*. The natural abundance of GVP as an agricultural waste and its high performance without requiring any treatment/modification indicate that it has good potential as an effective and sustainable biosorbent for Hg(II) removal.

## Data Availability

The data of the study are available on request.
